# Mechanistic Studies and Modeling Reveal the Origin of Differential Inhibition of Gag Polymorphic Viruses by HIV-1 Maturation Inhibitors

**DOI:** 10.1371/journal.ppat.1005990

**Published:** 2016-11-28

**Authors:** Zeyu Lin, Joseph Cantone, Hao Lu, Beata Nowicka-Sans, Tricia Protack, Tian Yuan, Hong Yang, Zheng Liu, Dieter Drexler, Alicia Regueiro-Ren, Nicholas A. Meanwell, Mark Cockett, Mark Krystal, Max Lataillade, Ira B. Dicker

**Affiliations:** 1 Departments of Virology, Bristol-Myers Squibb Research & Development, Wallingford, Connecticut, United States of America; 2 Pharmaceutical Candidate Optimization, Bristol-Myers Squibb Research & Development, Wallingford, Connecticut, United States of America; 3 Discovery Chemistry Platforms, Princeton, New Jersey, United States of America; 4 Discovery Chemistry, Bristol-Myers Squibb Research & Development, Wallingford, Connecticut, United States of America; 5 Global Clinical Development, Bristol-Myers Squibb Research & Development, Wallingford, Connecticut, United States of America; University of North Carolina at Chapel Hill, UNITED STATES

## Abstract

HIV-1 maturation inhibitors (MIs) disrupt the final step in the HIV-1 protease-mediated cleavage of the Gag polyprotein between capsid p24 capsid (CA) and spacer peptide 1 (SP1), leading to the production of infectious virus. BMS-955176 is a second generation MI with improved antiviral activity toward polymorphic Gag variants compared to a first generation MI bevirimat (BVM). The underlying mechanistic reasons for the differences in polymorphic coverage were studied using antiviral assays, an LC/MS assay that quantitatively characterizes CA/SP1 cleavage kinetics of virus like particles (VLPs) and a radiolabel binding assay to determine VLP/MI affinities and dissociation kinetics. Antiviral assay data indicates that BVM does not achieve 100% inhibition of certain polymorphs, even at saturating concentrations. This results in the breakthrough of infectious virus (partial antagonism) regardless of BVM concentration. Reduced maximal percent inhibition (MPI) values for BVM correlated with elevated EC_50_ values, while rates of HIV-1 protease cleavage at CA/SP1 correlated inversely with the ability of BVM to inhibit HIV-1 Gag polymorphic viruses: genotypes with more rapid CA/SP1 cleavage kinetics were less sensitive to BVM. *In vitro* inhibition of wild type VLP CA/SP1 cleavage by BVM was not maintained at longer cleavage times. BMS-955176 exhibited greatly improved MPI against polymorphic Gag viruses, binds to Gag polymorphs with higher affinity/longer dissociation half-lives and exhibits greater time-independent inhibition of CA/SP1 cleavage compared to BVM. Virological (MPI) and biochemical (CA/SP1 cleavage rates, MI-specific Gag affinities) data were used to create an integrated semi-quantitative model that quantifies CA/SP1 cleavage rates as a function of both MI and Gag polymorph. The model outputs are in accord with *in vitro* antiviral observations and correlate with observed *in vivo* MI efficacies. Overall, these findings may be useful to further understand antiviral profiles and clinical responses of MIs at a basic level, potentially facilitating further improvements to MI potency and coverage.

## Author Summary

HIV-1 continues to be a serious health threat, with nearly 40 million infected individuals worldwide. Despite effective treatment options, issues with resistance and drug toxicities illustrate the need for new drugs with novel mechanisms. Maturation inhibitors (MIs) block a key protease cleavage within its target, preventing formation of infectious HIV-1 virus. A first generation MI, (bevirimat), failed in clinical studies due to lack of broad spectrum activity, a result of amino acid polymorphisms around the site of action. BMS-955176 (GSK3532795) is a second generation MI active against these polymorphisms, and is currently in a Phase 2b study. We used a combination of antiviral and novel biochemical approaches to understand the mechanism for these spectrum differences. We find that while bevirimat exhibits incomplete antiviral activity, even at saturating drug concentrations, BMS-955176 exhibits greater ability to maximally inhibit these viruses, in part due to higher affinity for its target. These data were integrated into a semi-quantitative kinetic model whose outputs are in accord with in vitro antiviral observations and correlate with observed in vivo MI efficacies and the results of recent crystal and cryo-electron tomography structures. Our findings offer insights into MI activity and mechanism and may prove useful to help guide development of new MIs, with potential applicability to other virus systems and inhibitors.

## Introduction

Currently there are more than 1.2 million individuals (age 13 years older) in the United States (CDC data)[1] and more than 35 million worldwide infected with HIV, with 39 million people already having died from the disease and 2.3 million new cases reported in 2013.[2] There are presently >35 FDA-approved HIV therapies or combinations of agents which can be categorized into different classes: NRTIs, NNTRIs, PIs, integrase and entry inhibitors, (the latter includes attachment and fusion inhibitors, along with CCR5 antagonists).[3, 4] However, co-morbidities associated with long-term use of antiretrovirals (ARVs)[4–6] and the continued development of resistance remains a problem. [7, 8] Thus, there is a continuing need for new HIV-1 drugs which lack cross-resistance to existing classes and have excellent long term safety profiles.

HIV-1 maturation inhibitors (MIs) are a class of agents that may be effective in the treatment of HIV-1.[9–12] MIs disrupt the final step in the HIV-1 protease-mediated cleavage of the HIV-1 Gag polyprotein between capsid (CA) and spacer peptide 1 (SP1), a step which is responsible for a major conformational rearrangement of viral proteins within the virion that leads to the production of infectious virions.[13–15] The first generation HIV-1 maturation inhibitor, bevirimat (BVM), was halted in development[16] due to lack of clinical response in subjects whose viruses contained certain polymorphic Gag variants present in ~50% of the subtype B population, with such variations common among non-subtype B HIV-1 viruses.[17–27] Despite this result, BVM provided proof of concept (POC) in the clinic [28,29] that HIV-1 maturation inhibitors (MIs) *per se* might provide an effective alternative, should a next generation agent possess suitable pan-genotypic coverage.[30–32]

BMS-955176 (GSK3532795) was developed as a second generation MI that possesses antiviral activity against viruses containing BVM-resistant Gag polymorphisms.[9, 19, 23, 33–40] It is currently in Phase 2b clinical trials.[41–43] However, an understanding of the mechanism for how BMS-955176 achieves this improved antiviral coverage has not been described. Such an understanding at the mechanistic level is of intrinsic interest, potentially providing further insights into the maturation process itself, and the biology and biochemistry of HIV-1 infection. Of clinical importance, such understanding may also be of value to help guide the development of newer MIs with further improvements to MI activity, genotypic coverage and spectrum.

We took three approaches to address how BMS-955176 achieves these improvements to antiviral coverage. In the first, details of the antiviral dose-response profiles of BVM and BMS-955716 with respect to viruses containing various Gag polymorphs were studied. In a second approach, the mechanism of cleavage of capsid/spacer peptide 1 (CA/SP1) was evaluated using a novel LC/MS assay to quantitatively characterize the kinetics of cleavage HIV-1 Gag VLPs as a function of polymorph, while also determining the inhibitory effects of BVM and BMS-955176 in that system. Thirdly, the affinities and kinetics of dissociation of these MIs to these same Gag polymorphs in VLPs were measured using a radioligand binding assay.

Results reported herein indicate that reduced BVM antiviral activities toward certain polymorphs (elevated EC_50_ values) were accompanied by incomplete (less than 100%) inhibition of antiviral activity, even at saturating BVM concentrations. Thus, depending on polymorph, BVM may be described as a partial antagonist. On the other hand, BMS-955176 exhibits a significantly greater ability to maximally inhibit these Gag polymorphs. Biochemical characterization indicates that improvements to polymorphic coverage (both lower EC_50_s and higher degrees of maximal antiviral inhibition) are a result of its higher affinity for its target (Gag), which was shown to primarily be a result of its slower rate of dissociation. The antiviral and biochemical data herein reported were integrated into a model that calculates rates of CA/SP1 cleavage as a function of MI concentration and Gag polymorph, predicting *in vitro* antiviral profiles and estimating *in vivo* efficacy. These findings offer new insights into MI activity and mechanism and may prove useful to understanding the pre-clinical and clinical responses of MIs at a mechanistic level, potentially facilitating further improvements to newer MIs.

## Materials and Methods

### Cell lines and viruses

MT-2 cells were obtained from the NIH AIDS Research and Reference Reagent Program; 293T cells were obtained from the ATCC. Cell lines were sub-cultured twice a week in either RPMI 1640 (MT-2) or DMEM (293T) media (Gibco), supplemented with 10% heat inactivated fetal bovine serum (FBS, Gibco), and 100 units/mL penicillin with 100 μg/mL streptomycin (Gibco).

The parent WT virus was generated at Bristol-Myers Squibb from a DNA clone of NL_4-3_ obtained from the NIH AIDS Research and Reference Reagent Program[44] and contains the *Renilla* luciferase marker in place of viral *nef*, and the substitution of Gag P373 for serine, the most common B subtype variation at that position among B subtype viruses (NLRepRlucP373S). NLRepRlucP373S (WT) was modified to contain changes in Gag (for example, V362I, V370A, A364V, ΔV370,[40] the latter three of which encode high level resistance to BVM.[33, 40, 45] The recombinant viral DNA was then used to generate virus stocks by transfecting 293T cells (Lipofectamine PLUS kit, Invitrogen). Titers of all stocks were determined in MT-2 cells, using luciferase as the endpoint (Dual-Luciferase Reporter Assay System, Promega).[40, 46] The TCID50/ml (tissue culture infectious dose) was calculated by the method of Spearman-Karber.[47]

### Multiple cycle assay for the evaluation of antiviral susceptibilities

Compound susceptibilities of NLRepRlucP373S variants were examined using a multiple cycle infectivity assay as follows[40]: MT-2 cell pellets were infected with virus and re-suspended in cell culture medium. After a 1-hour pre-incubation at 37oC/CO2, cell-virus mixtures were added to a dose range of compound in 96-well plates at a final cell density of 10,000 cells per well. All compounds were tested at 1% final DMSO concentration. After 4–5 days of incubation at 37°C/5% CO2, virus yields were determined by *Renilla* luciferase activity (Dual-Luciferase Reporter Assay System, Promega) and the signals read using an Envision Multilabel Reader (PerkinElmer Product number: 2104). Maximal percent inhibition (MPI) values were calculated using the equation: MPI = (1- (signal from average at two highest drug concentrations/signal from no-drug control) * 100).

### Single cycle assay for the evaluation of single cycle MPI values

MI susceptibilities were also determined using an assay format similar to that reported. [36, 40, 48] which restricts viral growth to one replication cycle as follows: In a first step. 10 μg of the proviral clone of NLRepRlucP373S variant (containing the appropriate Gag substitution) and 8 μg of plasmid pSV-A-MuLV-env (MuLV envelope gene under control of the SV40 promoter, NIH AIDS Research and Reference Reagent Program, Cat# 1065) were co-transfected (calcium phosphate, Invitrogen, K2780-01) into 293T cells (60–70% confluence, T75 flask). After overnight incubation at 37°C/5% CO2, the transfected cells were washed, trypsin treated, and re-suspended in fresh medium at a density of 5 x 10^5^/mL. Cells were then distributed (100 μL/well) to 96 well plates that contained 100 μL of media with compound (compound was 3x serially diluted in DMSO, 1% final concentration of DMSO). In a second step, after 30 hours at 37°C/5% CO2, 100μL of supernatant (containing the newly produced virus) was transferred to a second 96 well plate to which fresh 293T cells (3x104/well) were added. Cultures were maintained for 2 days, after which cell-associated *Renilla* luciferase activity was measured upon the addition of Enduren (EnduRen Live Cell Substrate, Promega, catalog # E6485) and the signals read using an Envision Multilabel Reader (PerkinElmer Product number: 2104). MPI values were calculated as that compound concentration which inhibits 50% of the maximal signal (no-drug control) as described above. To demonstrate the late inhibitory phenotype of BMS-955176, the above single cycle assay was modified by the use of the HIV-1 envelope-deleted derivative pNLRepRlucP373 Δ*env*,[40] transfected with plasmid encoding HIV-1 LAI envelope (pLAIenv was constructed within BMS, contains the entire sequencing encoding LAI GP160 under control of the CMV promoter). LAI pseudotyped virus produced in a first step in the presence of inhibitor was added to MT-2 cells in the second step, instead of 293T cells as performed above.

### Preparation of HIV-1 Virus-Like Particles (VLPs)

HIV-1 virus-like particles (VLPs) are non-infectious particles that are made through transfection of a partial HIV-1 genome and contain only the Gag structural protein. VLPs used in these experiments [35, 36, 40] did not contain HIV-1 genes other than *gag*, and were prepared as follows: a synthetic gene (GagOpt)[49–51], under the control of the CMV promoter in plasmid 1_pcDNAGagOpt, was constructed to encode full length HIV-1 LAI Gag, with codons optimized for expression in mammalian cells. Various GagOpt clones were used, containing the coding sequence of LAI Gag or variant Gag polymorphs, starting from the N-terminus of matrix (MA, amino acid position 1) and extending to the stop codon of p6. The VLPs were produced[52, 53] by transfection (Mirus Bio LLC, TransIT®-LT1, cat# MIR 2300) of 293T cells (70–80% confluency in a T175 flask) with 18 μg of the appropriate pGagOpt plasmid. After 2 days of incubation at 37°C, supernatants (containing secreted VLPs) were cleared from cell debris by filtration (0.45-μm filter, Millipore #SCHVU01RE). The VLP particles were then pelleted through a 20% sucrose cushion at 25,000 rpm in an SW28 rotor for 2 hours, re-suspended in PBS at a total protein concentration of about 1000 μg/mL and then stored at -80°C.

### HIV-1 VLP Protease Cleavage Assay

Purified VLPs (~100 ng) were incubated at room temperature for 10–30 min in 10 μL of VLP buffer (50 mM MES pH 6.0, 100 mM NaCl, 2 mM EDTA and 2 mM DTT) supplemented with 0.06% Triton X-100 to remove the VLP lipid bilayer. Delipidated VLPs (~100 ng) were incubated with 3 μM MI (0.1% final DMSO) at 22°C for 2 hours, and then digested with HIV-1 protease by adding 1 μL of 2.7 μM of HIV protease (final concentration 0.27 μM, HIV-1 protease constructed to contain substitutions that limit auto-proteolysis: Q7K/L10I/I13V/L33I/S37N/R41K/ L63I/C67A/C95A)[54] One μL samples were taken at the indicated time points and digested with trypsin as follows: one μL of each HIV-1 protease digested sample was added to 24 μL of 50 mM ammonium carbonate (pH 8) containing 4 mM DTT. Samples were incubated at 60°C for 60 minutes, and then alkylated by the addition of 1 μL of 100 mM iodoacetamide. Samples were then kept in the dark for 30 minutes. Subsequently, 1 μL of 0.1 mg/mL reconstituted trypsin (Promega sequence grade modified trypsin, cat# 9PIV511) was added to each sample, and trypsin digestion was allowed to proceed at 37°C overnight. Reactions were stopped with 1 μL of formic acid, and peptides were analyzed by LC/MS. For MI inhibition studies MI (3 μM >500-fold antiviral EC_50_, 2 hour pre-incubation) MIs were first added to VLP to effect binding, after which time HIV protease was added to catalyze cleavage. Under these conditions the molar ratio of MI to Gag monomer is approximately 30-fold.

Liquid chromatography/mass spectrometry (LC-MS) analysis was performed using a Waters nanoAcquity UPLC system interfaced with a Thermo Scientific LTQ XL Orbitrap mass spectrometer affording nanoflow-LC/accurate mass data. Data were acquired by positive ion electrospray ionization using a Michrom Bioresources, Inc. Advance CaptiveSpray ion source operated at 1.5 kV and a transfer tube lens set at 150°C. Data on unique tryptic peptides of interest was acquired by single ion monitoring (SIM) using a 5 amu window in profile mode at a resolution of 7500 @ ½ ht. NanoLC analysis was carried out using a Waters Symmetry C18 180 μm x 20 mm 5 um (PN-186003514) trap column and a Microm Magic C18AQ 0.1 x 150 mm (PN-CP3/61271/00) analytical column. Trapping was performed at 5 μl/minute for 2 minutes at the initial gradient composition prior to the analytical gradient. The mobile phase composition was water (MP-A) and acetonitrile (MP-B) with each containing 0.1% formic acid. The analytical gradient was as follows 5%B to 35% MP-B over 30 min (ramped to 70% MP-B followed by equilibration at 5% MP-B) at a flow rate of 500 nL/minute. The mobile phase composition was water (MP-A) and acetonitrile (MP-B) with each containing 0.1% formic acid. Three microliters injection volumes were used for each sample. Data was analyzed using Thermo Xcalibur Processing software 3.0.63 and Thermo Xcalibur Quanbrowser software 3.0.63. Areas were measured using the plus 2 charge state mono isotopic mass of the peptides of interest +/- 0.2 amu from the peak apex.

The raw peak area for the SQ peptide (SLFGNDPSSQ, internal trypsin cleavage fragment at the C-terminal terminal end of Gag), was used as an internal control for normalization of the response for the peptides of interest. The data for the AM peptide (Gag SP1, generated by HIV-1 Pr cleavage at the N- and C-termini of SP1), the VM peptide (generated by HIV-1 Pr cleavage between SP1 and nucleocapsid, then cleavage by trypsin) and the VR peptide (cleavage by trypsin only, no internal cleavage by HIV-1 Pr) were normalized against the data from the SQ peptide. The percent of total = 100 x [AM/SQ / (AM/SQ + VM/SQ + VR/SQ)], where AM/SQ + VM/SQ + VR/SQ = the sum of all the peptide fragments encompassed within the two trypsin cleavage sites on either side of the SP1 peptide.

### Specific Binding of MI to HIV-1 Gag VLPs

Specific binding of MIs to VLPs were determined using a scintillation proximity (SPA) radiolabeled binding assay. VLPs (0.5 to 1.2 μg in PBS) were mixed with 100 μg of SPA beads (PBS suspension, PVT WGA SPA beads, PerkinElmer, cat # RPNQ0250) in 40 μL of total volume per well (96-well plate, Corning, white low binding, cat# 3600) After 1-hour incubation at room temperature, the volume was increased to 180 μL /well by the addition of binding buffer (100 mM Tris, pH 6.5, 2 mM EDTA, 0.03% Tween-20, 5 mM MgCl_2_). The final concentration of DMSO in the assay was 10% by volume. For determination of K_d_ values by a competition method, 20 nM of [^3^H]-BMS-977660 (a C:20 double bond reduced (tritiated) form of BMS-955176) [35, 40] was added to the VLP/bead mixtures, to which was added a serial dilution (0.04–3000 nM) of non-radiolabeled MI. After 4 hour-equilibration at room temperature, bound [^3^H]-BMS-977660 was measured using a Top Count plate reader (PerkinElmer). The data were fit to an equation for heterologous competition (GraphPad v 5.1).

### Kinetics of MI dissociation from HIV-1 Gag VLPs

MI dissociation rates were measured by adding 30 nM [^3^H]–BMS-885221 (a C:20 double bond reduced form of BVM) or 20 nM [^3^H]–BMS-977660 to SPA bead/VLP (0.5 to 1.2 μg) complexes, allowing binding to reach equilibrium for 3 hours at room temperature. After this time a 40-fold molar excess of unlabeled competitor MI was added to effect irreversible displacement of the [3H] MI. Kinetics of disappearance of the bound 3H MI were monitored using a Microbeta2 plate reader (PerkinElmer) and the data fitted to a first order exponential equation (GraphPad Prism v5.1).

### Model for inhibition of CA/SP1 cleavage (inhibition of infectivity) by MIs

Details of the model are shown in later in the Results section. In the presence of an MI, the rate of CA/SP1 cleavage, and thus the formation of mature virus, is derived below. Since the measured dissociation rate constants of the MIs (*k*_*off*_) are faster than the innate rates of CA/SP1 cleavage (*k*_*1*_) for the WT and polymorphic viruses, a rapid equilibrium assumption was employed to derive the observed rate constant (*k*_*clv*,*ob*_) to form mature virus (C). With this assumption, the association and dissociation rates of MI binding are the same.

$$k_{off}\left[A\right]=k_{on}\left[MI\right]\left[B\right]\;\mathrm{or}\;\left[A\right]=\frac{\left[MI\right]\left[B\right]}{K_{d}}\;(\mathrm{since}\;K_{d}=\frac{k_{off}}{k_{on}})$$ (1)

The concentration of total immature virus equals the sum of free immature virus (B) plus the MI bound immature virus (A) and is defined as Im_total_.

$$\left[{Im}_{total}]=[A]+[B\right]$$ (2)

Replacing [A] in Eq 2 from Eq 1:

$$\left[{Im}_{total}]=\frac{\left[MI][B\right]}{K_{d}}+[B\right]$$ (3)

Rearranging Eq 3, the relationship between B and Im_total_

$$\left[B]=\frac{K_{d}}{[MI]+K_{d}}[{Im}_{total}\right]$$ (4)

The formation of mature virus (C), and the depletion of total immature virus (Im_total_) have the same rate, thus

$$\frac{d[C]}{dt}=-\frac{d[{Im}_{total}]}{dt}=k_{1}[B]+k_{2}[A]=k_{1}[B]+k_{2}\frac{\left[MI\right]}{K_{d}}[B]$$ (5)

Substituting [B] in Eq 5 with Eq 4:

$$\frac{d[C]}{dt}=-\frac{d[{Im}_{total}]}{dt}=(\frac{k_{1}\;*\;K_{d}}{[MI]+K_{d}}+\frac{k_{2}\;*\;[MI]}{[MI]+K_{d}})[{Im}_{total}]$$ (6)

Integrating Eq 6, the solution of equation for the cleavage of CA/SP1, and thus formation of mature virus (C) is

$$Ln\frac{\left[C\right]}{\left[T\right]}==(\frac{k_{1}*K_{d}}{[MI]+K_{d}}+\frac{k_{2}*[MI]}{[MI]+K_{d}})t$$ (7)

where t is the time, assuming at t = 0 there is no cleaved CA/SP1 or mature virus existing, and T is the total concentration virus.

The observed rate constant (*k*_*clv*,*ob*_) to form capsid from CA/SP1, and thus mature virus, in the presence of MI is:

$$k_{clv,ob}=\frac{k_{1}*K_{d}}{[MI]+K_{d}}+\frac{k_{2}*[MI]}{[MI]+K_{d}}$$ (8)

## Results

### Second generation MI, BMS-955176, has potent antiviral activity toward naturally occurring HIV Gag variants which are resistant to bevirimat

**Fig 1:**
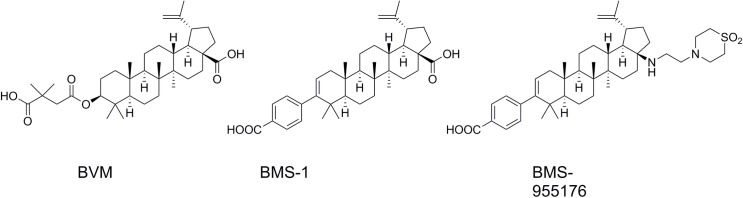
Structure of MIs used in this study.

Previous reports indicated that a first generation MI, BVM, demonstrated poor antiviral activity both preclinically and in a POC study toward clinical isolates[28, 29] containing polymorphic substitutions in Gag around the site of its mechanism of action, i.e., at or near the HIV-1 protease-mediated cleavage site between capsid (p24) and spacer peptide 1[9, 19, 20, 23, 33, 45] These polymorphs include substitutions at Gag positions V362, Q369, V370 and T371.[19–21] BMS-955176 was identified as a clinical candidate with improved potency against viruses containing these polymorphic substitutions, low human serum binding and excellent PK properties [35–38, 40, 55] BMS-955176 (Fig 1) retains potent activity toward these polymorphic variants *in vitro* and was active in a Ph2a POC study[41–43] As shown in Table 1,[40] BMS-955176 is 5.4-fold more potent than BVM toward WT virus, and polymorphic viruses retain sensitivity to BMS-955176, with FC values (EC_50_/WT EC_50_) between 1- and 6.8-fold.[35, 36, 40] The protease inhibitor nelfinavir was used as a control, which exhibits similar antiviral characteristics towards all the polymorphic viruses. By comparison, BVM exhibits significantly reduced activity toward these variants (up to >1000 fold). For example, BMS-955176 retains activity toward variants with substitutions at Gag V370 by alanine or methionine (1.4- and 1.5-fold, respectively), and V362I (2.4-fold), as compared to 54-, 177- and 7.2-fold losses of sensitivity by BVM, respectively. In addition, BMS-955176 retains activity toward virus with V370A/ΔT371 and ΔV370 substitutions, both minor polymorphs in subtype B, but characteristic of subtype C isolates[24] (FCs of 3.5 and 6.8-fold, respectively). By comparison, BVM is >100-fold less active toward both V370A/ΔT371 and ΔV370-containing viruses. An early BMS compound in the series leading to the identification of BMS-955176 was BMS-1 (Fig 1),[37] with an antiviral profile similar to BVM. It was included in this study to determine if the results were able to be generalized beyond BVM and BMS-955176. BMS-955176 does not inhibit A364V[40], a resistance mutant selected for by BVM in vitro[33] and also reported in two HIV-1 subjects in a clinical trial with BVM.[56]

**Table 1:** Antiviral Activity of MIs Toward Gag Polymorphic Viruses

Virus	NFV		BVM		BMS-1		BMS-955176	
	EC_50_	FC	EC_50_	FC	EC_50_	FC	EC_50_	FC
WT	4.3	1.0	10.2	1.0	16	1.0	1.9	1.0
V362I	4.3	1.0	74	7.2	213	14	4.5	2.4
Q369H	n.d.	n.d.	7.0	0.7	n.d.	n.d.	1.9	1.0
V370A	4.1	1.0	552	54	233	15	2.7	1.4
V370M	4.5	1.0	1810	177	>4000	>250	2.8	1.5
ΔV370	5.2	1.2	>10000	>1000	>6000	>375	13	6.8
V370A/Δ371T	5.7	1.3	1114	109	>10000	>625	6.6	3.5
T371A	n.d.	n.d.	40	3.9	28	1.8	2.0	1.1
ΔT371	2.0	0.5	77	7.5	292	19	7.3	3.8
A364V	5.1	1.2	>10,000	>10000	>10,000	>625	1480	759

EC_50_ values (nM) as assayed with the HIV-1 laboratory strain NLRepRlucP373S containing the indicated site directed polymorphic substitutions (SDMs); FC = EC_50_ (SDM)/EC_50_ (WT), data from multiple measurements (N ≥ 3); nd = not determined. The data provided are the mean of at least triplicate values with a mean CV of 83 +/- 50% for all the experiments, and all CV values < 200%. n.d. = not determined.

Overall, these results indicate that BMS-955176 exhibits significantly improved *in vitro* antiviral activity toward polymorphic variations in Gag which result in reduced sensitivity to first generation MIs. With these results in hand we initiated virological and biochemical studies whose aim was to understand the mechanistic basis for the improved antiviral profile of BMS-955176.

### Incomplete antiviral inhibition of polymorphic Gag viruses is characteristic of first generation MIs

**Fig 2:**
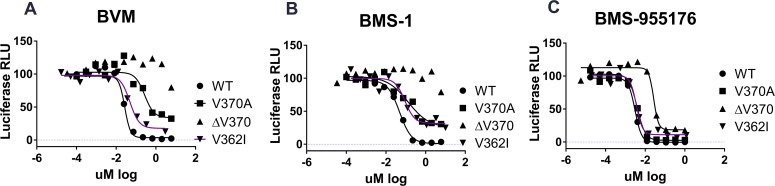
Representative antiviral dose-response plots for selected MIs in a multiple cycle antiviral assay. Representative examples of antiviral inhibition curves in a multiple cycle replication assay for A) BVM, B) BMS-1 and C) BMS-955176 toward WT, V362I, V370A and ΔV370 viruses. Maximal percent inhibition (MPI) values were calculated from the plateaus in inhibition at the highest inhibitor concentration, and tabulated and normalized for multiple experiments in Table 2 using the equation MPI = (1 - (signal from the average at the two highest drug concentrations/signal from no drug control) * 100). Units are in *Renilla* luciferase relative light units (RLU). In cases where there is no curve, and thus no plateau *per se*, the same calculation method was employed using the two values at the highest compound concentration (6 μM).

Earlier biochemical studies had noted that while BVM disrupts the final step of HIV-1 maturation, that of CA/SP1 processing, this disruption is not an absolute block: some mature CA is generated, even at high concentrations of the compound.[57] We considered it possible that partial biochemical inhibition might translate into partial inhibition in antiviral assays. This concept was evaluated by conducting detailed studies of the antiviral inhibition dose response curves of BVM toward less susceptible Gag polymorphs, focusing on the degree of inhibition at the highest BVM concentrations tested. Initial studies made use of a multiple cycle (MC) infectious virus assay using HIV-1 luciferase reporter viruses. In this format, a low viral input (multiplicity of infection typically 0.002–0.005) was used, and therefore multiple rounds of virus release and viral re-infection were required to achieve sufficient luciferase signal for detection at the assay endpoint (typically 4 days post infection). The results are shown in Fig 2 and Table 2. In this format, BVM inhibition of the control WT virus (percent maximal inhibition (MPI +/- Stdev) of 98.4 +/- 1.0) was similar to that achieved by NFV, an HIV protease inhibitor used as a positive control ((percent maximal inhibition (MPI) of 98 +/- 0.9 compared to 100%, respectively). By comparison, BVM inhibition of the polymorphic viruses V362I and V370A reproducibly achieved only partial inhibition (MPI values of 81.3 +/- 2.0 and 65.4 +/- 3.8, respectively). Improvement to the MPI of the V362I and V370A viruses was not achieved at higher BVM concentrations, as a plateau in inhibition at a concentration occurred at approximately 1 μM. Representative examples of dose response curves are shown in Fig 2 for BVM, BMS-1 and BMS-955176. The ΔV370 virus variant is resistant to BVM, exhibiting an MPI of 9.2+/- 6.3 (Table 2). Control experiments performed with 3 and 6 μM MI dissolved in 10% FBS media vs. PBS buffer for up to 4 days, found that upon subsequent evaluation the concentration of MI between 80–100% in the media, indicating no loss due to precipitation of MI under these conditions. Average recovery in the PBS condition was ~50%, indicating precipitation and binding to the walls of the tube (S1 Fig). This result indicates that the plateau in inhibition is not an artifact of limited MI solubility under the cell culture conditions. Secondly, as discussed later below, (behavior of BMS-955176 toward the ΔV370 virus), there is an obvious plateau in inhibition at 100 nM in a single cycle assay of approximately 50% of maximal, but in a multiple cycle assay the maximal percent inhibition (MPI) is higher (91.9%). Such pronounced plateaus were observed in other single cycle assays (see values in Table 2). If solubility were to be the limiting factor, both single and multiple cycle formats would be expected to provide similar plateaus.

**Table 2:** Maximal Percent Inhibition from Multiple Cycle (MC) and Single Cycle (SC) Assays

	MC Assay MPI (%)												SC Assay MPI (%)								
	WT			V362I			V370A			ΔV370			WT			V370A			ΔV370		
MI	MPI	SD	N	MPI	SD	N	MPI	SD	N	MPI	SD	N	MPI	SD	N	MPI	SD	N	MPI	SD	N
BVM	98.4	1.0	8	81.3	2.0	4	65.4	3.8	4	9.2	6.3	4	82.3	2.7	15	19.0	3.5	3	-25.6	30.9	12
BMS-1	97.0	0.5	4	71.4	5.4	4	63.2	4.7	3	3.7	2.9	3	nd	nd	nd	nd	nd	nd	nd	nd	nd
BMS-955176	100	0.4	11	93.8	1.8	4	98.2	1.3	6	91.9	4.4	6	93.0	2.5	26	76.5	3.0	5	45.9	7.4	30
NFV	100	0.2	10	100	0.3	5	99.9	0.1	5	100	0.1	5	99.3	0.4	21	99.0	0.6	4	99.1	0.9	26

BVM = bevirimat, MPI = maximal percent inhibition, MC = multiple cycle, SC = single cycle. nd = not determined.

BMS-1, an early compound in the development of the structure activity relationship (SAR) leading to the identification of BMS-955176, shares structural similarity (Fig 1) to both BVM and BMS-955176 but differs from BVM by replacement of the C3 dimethylsuccinic acid by a benzoic acid.[37] Similar to BVM, the first generation BMS-1 (FC values similar to BVM, Table 1) exhibits a high MPI against wild type virus (97.0 +/- 0.5), but only partially inhibits V362I and V370A viruses (MPIs = 71.4 +/- 5.4 and 63.2 +/- 4.7, respectively) and does not inhibit ΔV370 containing virus (MPI = 3.7 +/- 2.9) (Fig 2B and Table 2). By comparison, BMS-955176 exhibits high MPI values of 100 +/- 0.4, 98.2 +/- 1.3, 93.8 +/- 1.8 and 91.9 +/- 4.4 for WT, V370A, V362I and the ΔV370 containing viruses, respectively (Fig 2C and Table 2).

**Fig 3:**
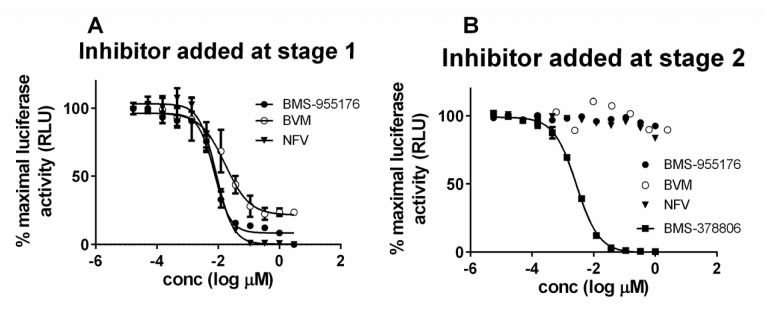
Antiviral profiles of selected ARVs of differing mechanism of action in the SC Assay. A) Test compound added during transfection to produce LAI pseudotyped NLRepRlucP373 Δ*env* and evaluated for activity by infections in stage 2, as described in text; B) Virus produced by transfection, test compound added at the second stage of virus infection, as described in text; BMS-955176 (MI, filled circles); BVM (MI, open circles); nelfinavir (protease inhibitor, triangles), BMS-378806 (HIV attachment inhibitor, squares).

To further probe the phenomenon of incomplete inhibition of various polymorphic viruses, we employed a 2-step single cycle assay in which HIV-1 LAI pseudotyped virus is first released into the supernatant by co-transfection of NLRepRlucP373Δ*env* and pLAI envelope plasmids into 293T cells in the presence of MI.[40] Subsequently, the supernatant is harvested and used for infection of MT-2 cells in a second infection step. In a manner similar to the Magi assay[48, 58] a signal in the second infection step indicates that infectious virus had been produced in the transfection step. However, subsequent rounds of infection are prevented as virus produced in the second stage lacks an HIV-1 envelope, and is thus unable to infect MT-2 cells. An inhibitor which blocks the production of infectious virus in the transfection stage of the assay will score as inhibitory in the second stage of the assay. Since this assay monitors the events that have taken place in a single cycle of infection, we refer to this format as a single cycle assay, or SC assay. Control experiments established that when a late inhibitor such as nelfinavir is added at the transfection step, luciferase activity is inhibited in the infection stage (Fig 3A). However, when NFV is added only at the infection stage, luciferase production is not inhibited (Fig 3B). The HIV-1 attachment inhibitor, BMS-378806[59] is fully active (Fig 3B), as expected for an agent which inhibits early in the HIV-1 life cycle. The MIs BVM and BMS-955176 behaved similarly to nelfinavir, inhibiting luciferase production only when added in the first step of the assay, consistent with their late mechanism of action.

**Fig 4:**
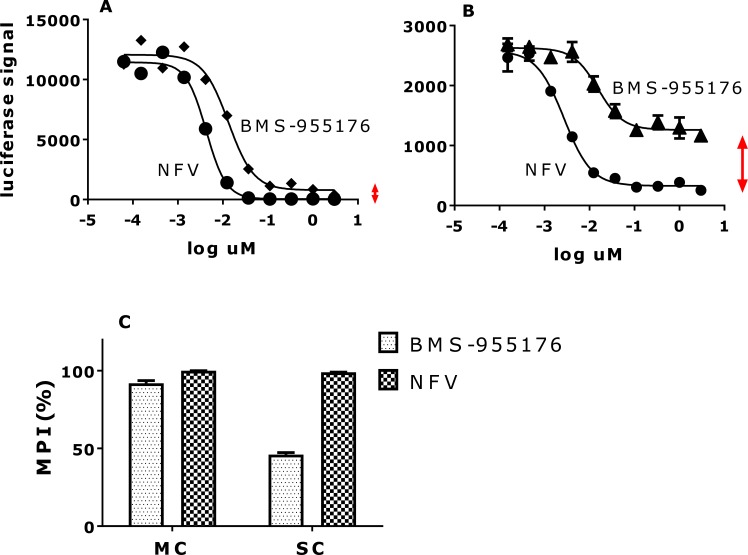
Comparison of antiviral inhibition profiles of the ΔV370 virus by BMS-955176 in MC and SC assays. Dose-response curves for BMS-955176 (diamonds) and NFV control (circles) in the A) MC and B) SC assay toward the NLRepRlucP373ΔV370 virus; vertical double-headed arrows highlight differences in residual signal (100-MPI) between the two compounds in the two antiviral formats, C) comparison of MPI data for A and B. Data shown an average of 3 experiments.

As shown in Table 2, BVM exhibited an SC MPI value towards WT virus of 82.3 +/- 2.7, less than BMS-955176 in this more demanding format, while BVM barely inhibited the V370A virus (MPI of 19.0 +/- 3.5), a result which is qualitatively similar to that obtained using the MC format. The ΔV370 variant is resistant to BVM in this assay. In contrast, BMS-955176 exhibits SC MPI values of 93.0 +/- 2.5, 76.5 +/- 3.0 and 45.9 +/- 7.4 towards the WT, V370A and ΔV370 viruses, respectively (Table 2). Overall, MPI values in both the SC and MC formats follow the same trend, but SC MPI values are reproducibly lower, presumably due to the fact that viral challenge is higher in the transfection format vs. infection (MC, low multiplicity of infection = 0.005), and the absence of multiple cycles which inhibit breakthrough virus from within each preceding cycle. Antiviral dose response curves for inhibition of the ΔV370 virus by BMS-955176 are compared using the two formats (MC assay, Fig 4A and SC assay, Fig 4B). Fig 4C shows the differences in MPI values from Fig 4A and 4B, where the control NFV exhibits full inhibition in both formats. BMS-955176 inhibition of ΔV370 in the MC assay did not reach the 100% control value of NFV (Table 2, Fig 2C). The single cycle assay provides a wider dynamic range from which to understand the nature of the stable incompletely inhibited plateau, as compared to the multiple cycle assay (~100 nM BMS-955176 toward ΔV370 (Fig 2B)).

### Model for quantifying inhibition of CA/SP1 cleavage by MIs

**Fig 5:**
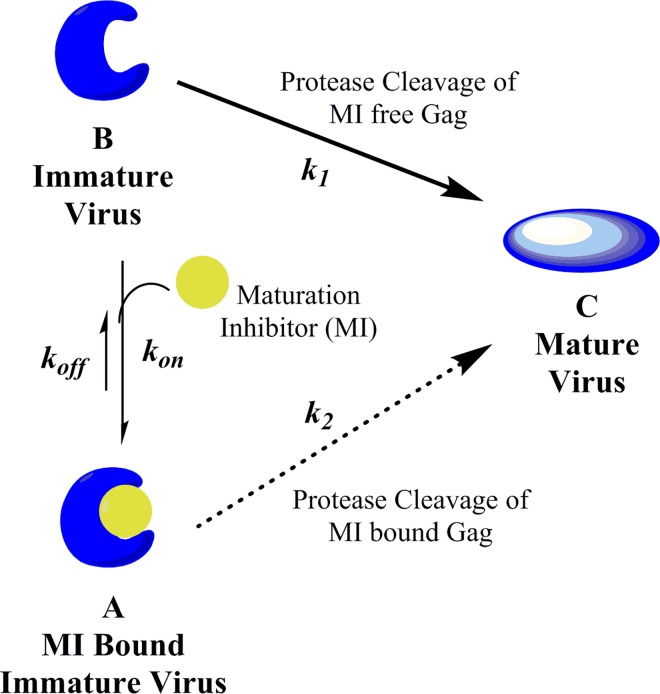
Scheme for inhibition of HIV-1 infectivity by MIs. Models that depict the inhibition of mature virus formation by HIV-1 maturation inhibitors. Immature virus B is in equilibrium for binding to MI to produce MI-bound immature form A. In model 1, HIV protease can only cleave immature virus (form B) in the absence of MIs. The innate cleavage rate constant (*k*_1_) is a function of Gag polymorphs. In model 2, HIV-1 protease may cleave CA/SP1 in either form A (bound) or form B (unbound) to produce mature virus C, thus the former pathway provides an escape mechanism for the formation of mature virus in the presence of MIs with rate constant *k*_2_. This second cleavage rate constant (*k*_2_) is a function of both MIs and Gag polymorph, and is derived from inclusion of MPI values from multiple (model 2a) or single (model 2b) cycle antiviral assays (Table 2).

To understand the partial antiviral inhibition results we considered the basic framework for the underlying mechanism of maturation inhibition, i.e. its capacity to block the last cleavage step during virion biogenesis, that of CA/SP1 cleavage by HIV-1 protease.[30, 31] As depicted in Fig 5, maturation inhibitor (MI) binds to the immature HIV Gag polyprotein in the vicinity of the cleavage site[60–62] to produce the MI-bound form (A), in which CA/SP1 is protected from HIV-1 protease cleavage. As reported, action of MIs on Gag VLPs requires that Gag be fully assembled in its quarternary state, [9, 63] in concordance with this we have observed that heat inactivation of VLPs abrogates specific MI binding. Binding is reversible,[35, 40] with association and dissociation rate constants defined as *k*_*on*_ and *k*_*off*_ respectively. The innate cleavage rate constant (*k*_*1*_) determines the efficiency of the irreversible conversion from immature virus (B) to mature virus (C). Based on the observed maximal percent inhibition (MPI) values from the cellular antiviral assays, we hypothesize that the MI-bound immature virus (A) can also be cleaved by HIV-protease, but at a reduced rate (*k*_*2*_, *where k*_*2*_ *< k*_*1*_), thus accounting for the production of virus, as a function of polymorph and MI, even at saturating concentrations of MI.

In this model (derivation in Materials and Methods) *k*_*1*_ is specific for each Gag polymorphism, while *k*_*2*_ is a function of both MI and Gag polymorphism. Thus, mature virus C will be produced as a function of time in a manner dependent on the steady state concentrations of both the free immature form B and the MI-bound immature form A, and dependent on their respective rate constants, *k*_*1*_ and *k*_*2*_, for HIV-1 protease cleavage of CA/SP1. To challenge this scheme and model this process, we created appropriate biochemical assays needed to obtain the requisite protease CA/SP1 cleavage rate constants and MI affinities toward the WT and polymorphic variant viruses.

### CA/SP1 of Gag VLPs from BVM resistant viruses is cleaved more rapidly than WT

**Fig 6:**
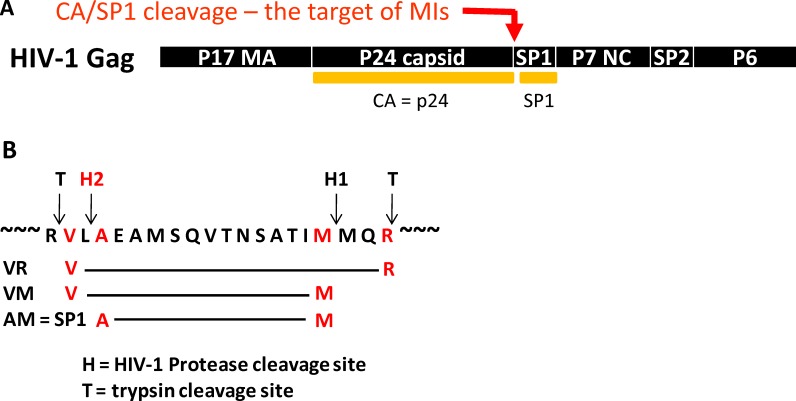
Schematic for Cleavage of CA/SP1. A) Schematic for the processing HIV Gag at CA/SP1 and SP1/NC sites by HIV-protease. B) Detail of the cleavage region around CA/SP1 showing sites for HIV-1 protease cleavage (H1 and H2) and sites for subsequent cleavage by trypsin (T).

An LC/MS analysis method to quantify the specific event inhibited by MIs was developed that measured the HIV-1-mediated cleavage of CA/SP1 (p25) to CA (p24) and SP1 through quantitation of a peptides released after subsequent trypsin cleavage (Fig 6A).[35]

This method entails exposure of HIV-1 Gag virus-like particles to HIV-1 protease *in vitro* in the absence or presence of MIs, followed by trypsin cleavage of the resulting HIV-1 protease-mediated products. The starting parental material (no cleavage at either SP1/NC or CA/SP1) is referred to as peptide VR, by virtue of the N- and C-terminal amino acids of the peptide produced by trypsin cleavage of Gag (Fig 6B). Cleavage by HIV-1 protease between SP1 and NC at site H1, and subsequent cleavage by trypsin, produces intermediate peptide VM (Fig 6B). The N-terminal valine of VM is derived from trypsin cleavage while the C-terminal methionine is derived from HIV-1 protease cleavage. Lastly, HIV-1 protease cleavage at CA/SP1 at HIV-1 protease site H2 produces peptide AM (Fig 6B, peptide nomenclature as further described in Materials and Methods, peptide AM = Gag peptide SP1).

**Fig 7:**
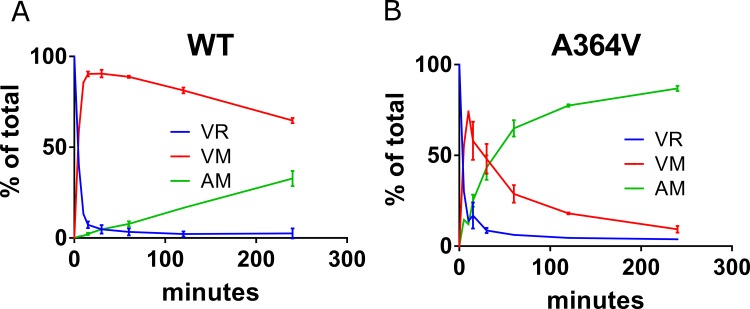
Cleavage of CA/SP1. A) Average of two experiments showing kinetic cleavage profiles for WT and B) A364V HIV-1 Gag VLPs; VLPs and HIV-1 protease concentrations = 0.1 mg/mL and 270 nM, respectively. AM = SP1 peptide; further details in Materials and Methods.

This method is suitable for monitoring the 3 species simultaneously, allowing for measurement of the kinetics of cleavage at both CA/SP1 and NC/SP1 (representative experiments for wt and A364V are shown in Fig 7C and 7D, respectively).

In the example of Fig 7C (wt), an average of two independent experiments at an HIV-1 protease concentration of 270 nM, the parent peptide VR disappears first due to rapid cleavage between SP1 and NC, as has been reported.[13–15] Rapid disappearance of VR is accompanied by the release of intermediate VM (VM is essentially the surrogate for p25), which decreases as AM (SP1) is formed. AM (SP1) peptide appears slowly, gradually increasing with time, but its formation remains incomplete at the last time point (240 minutes) under this set of conditions. By comparison, for A364V (Fig 7D) the disappearance of parent VR is similarly rapid vs. wt, while the appearance of intermediate VM, and product AM (SP1) are faster than wt.

Measured rate constants (k_*clv*,*ob*_ at 270 nM HIV-1 protease) for the cleavage of CA/SP1 by HIV-1 protease at CA/SP1 from WT and BVM-resistant polymorphic VLPs are shown in Table 3. For comparison to the cleavage rate data, multiple cycle antiviral sensitivities from Table 1 are also shown in this table. As might be expected, absolute cleavage rates were a function of the HIV-1 protease concentration; they were linear over the range of 67–540 nM HIV-1 protease (S2 Fig), indicating no loss of proteolytic activity within this time window, as expected for use of protease specifically engineered to not undergo autoproteolysis.[54] CA/SP1 cleavage of WT VLPs was the slowest, while VLPs containing V370A and V362I were cleaved approximately 3-fold faster than WT (Table 3). The subtype C-like surrogate polymorphic VLPs, V370A/ΔT371 and ΔV370, were cleaved 2.2- and 2.7-fold faster than WT. By comparison, A364V, the completely BVM and BMS-955176-resistant variant, [9] was cleaved ~10-fold faster than WT, as reported.[64] A set of representative AM peptide (SP1) appearance curves is shown in S3 Fig: the order of appearance of SP1 product is A364V > V370A, V362I, ΔV370 > V370A/ΔT371 > WT, which is a similar, but in inverse order, to the antiviral sensitivities of these polymorphic viruses to BVM (Tables 1 and 2).

**Table 3:** Rates of cleavage of WT and Gag polymorphic VLPs at CA/SP1 by HIV-1 protease *in vitro*

	Biochemical cleavage		Antiviral FC-EC50	
Virus	k_clv, obs_ min^-1^ ^a^	Rel k_*clv*,*ob*s_ (*vs*. WT)^a^	BVM	BMS-955176
WT	0.0015	1.0	1.0	1.0
V370A/ΔT371	0.0032	2.2	109	3.5
ΔV370	0.0039	2.7	>1000	6.8
V362I	0.0043	2.9	7.2	2.4
V370A	0.0045	3.0	54	1.4
A364V	0.014	9.7	>1000	759

^a^[HIV-1 protease] = 270 nM, 22°C. FC data from Table 1; FC_:_ antiviral using the MC assay, FC-EC50 = EC_50_ SDM/EC_50_ WT; WT = NLRepRlucP373S; cleavage data are an average of values performed in triplicate with CVs of ~20% between experiments.

HIV-1 protease specifically designed to inhibit auto-proteolysis was used,[54] as initial experiments of wt HXB2 HIV-1 protease produced unsatisfactory results in terms of non-linearity of cleavage with time. As can be seen in S2 Fig, there is linearity of cleavage for wt for concentrations of protease up to 540 nM, an indication of no loss of proteolytic activity, with the kinetic data reported in Table 3 performed using 270nM protease. There was non-linearity for A364V cleavage at 270nM protease at longer time points, thus the rate constant data for A364V was derived from within the linear range only. A sub-analysis of the rates in the linear range over multiple concentrations of protease indicated that the relative 2^nd^ order rate constant for A364V (S2 Fig) is 9-fold faster than wt, in agreement with the 1^st^ order constant, and indicating that the first order rate constant accurately captures this information. The relative rate of cleavage of A364V (9.7-fold) is in accord with a value previously published (7.6-fold).[64]

### BMS-955176 more efficiently inhibits *in vitro* CA/SP1 cleavage *vs*. BVM

**Fig 8:**
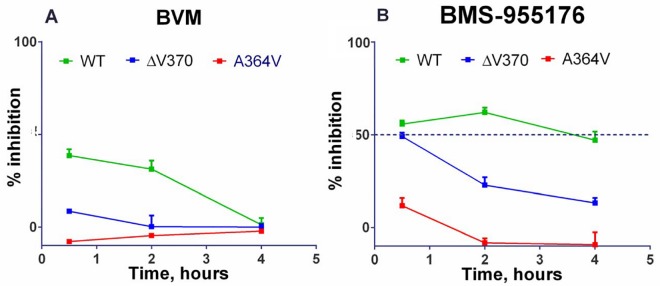
Inhibition of HIV-1 protease mediated CA/SP1 cleavage by MIs. A) BVM, B) BMS-955176: Inhibition of CA/SP1 cleavage of WT, ΔV370 and A364V VLPs *in vitro* as monitored by LC/MS analysis (Materials and Methods); values are an average of 9 replicates; bars are SEM; MI concentrations: 3 μM.

BVM and BMS-955176 were evaluated for their abilities to inhibit CA/SP1 cleavage of the Gag polyprotein using the LC/MS analysis method. Preliminary experiments established that MI binding to VLPs reached equilibrium within 2 hours, so incubations with MI were maintained, prior to adding protease. As shown in Fig 8A (left panel), 3 μM BVM or BMS-955176 inhibit the production of final product AM (SP1) from wt VLPs. In addition, inhibition of cleavage data by the MIs are not due to non-linear rates of cleavage, due artifactually from loss of proteolytic activity, but rather, are due to innate differences in cleavage rates (see above, protease engineered to limit autoproteolysis and cleavage rates calculated from within the linear range).

However, BVM inhibition of WT CA/SP1 cleavage was not maintained throughout the entire time course of the cleavage experiment, as it dropped from 39% inhibition at 2 hours to 1% inhibition at 4 hours (Fig 8A). On the other hand BMS-955176 exhibited sustained inhibition over the 4 hour period with WT VLPs. This persistence of *in vitro* CA/SP1 cleavage inhibition trended with the antiviral cell culture MPI values (Table 2). For example, the sustained inhibition of cleavage of WT CA/SP1 by BMS-955176 correlates to its single cycle MPI value of 93% (100% for multiple cycle MPI) towards WT virus in cell culture, whereas the loss in *in vitro* inhibition of CA/SP1 cleavage at longer time points by BVM towards WT correlated to its single cycle MPI of 82% (98% for multiple cycle MPI).

VLPs containing the ΔV370 polymorphism were also evaluated in this assay. BMS-955176 inhibited ΔV370 cleavage to a degree similar to BVM inhibition of WT at the earliest time point (30 minutes) and did exhibit time-dependence, but the loss of inhibition was slow, with ΔV370 cleavage still remaining partly inhibited (13%) at the 4 hour time point. BVM was not inhibitory at any time point toward ΔV370 containing VLPs. Again, the time-dependent inhibition in this assay correlates to lower MPI values in cell culture with BMS-955176/ΔV370 values of 46% for its single cycle MPI (92% for multiple cycle MPI) and BVM/ΔV370 values of -26% (SC MPI) and 9% (MC MPI). Interestingly, while BVM did not inhibit cleavage of A364V, BMS-955176 reproducibly exhibited a small degree (~10%) of inhibition at the earliest time point, but was not inhibitory by 2 hours (Fig 8B). Multiple cycle MPI values for both compounds against A364V containing virus were near zero.

Thus, lower MPI values are correlated to both reduced antiviral potency (elevated MC EC_50_s) and a time-dependent loss of *in vitro* inhibition of CA/SP1 cleavage. Conversely, higher antiviral MPI values are correlated with greater antiviral potency (lower MC EC_50_s) and correlated with persistence of *in vitro* inhibition of CA/SP1 cleavage over time.

### BMS-955176 binds to VLPs containing Gag polymorphs with higher affinity and dissociates more slowly than first generation MIs

To complete the data required to model MI inhibition of CA/SP1 cleavage (Fig 5) as a function of MI and Gag polymorph, specific binding affinities of BVM, BMS-1 and BMS-955176 toward VLPs containing Gag polymorphs were determined through the use of a competitive radioligand binding assay (Table 4).[40] Examples of competition displacement assay results are provided in S4 Fig, including BMS-955176/A364V. BMS-955176 affinity for WT Gag VLPs was 3.2 nM, with slightly lower affinity for V362I (4.3 nM), and reduced affinity (2- and 10-fold) for V370A and ΔV370 VLPs, respectively. By comparison, BVM affinity toward WT was 5.4 nM, which was reduced 2.9-, 9.1- and 48-fold toward V362I, V370A and ΔV370, respectively. BMS-1 affinities were 3-5x reduced, as compared with BVM. The binding of BMS-955176 toward A364V was measurable (K_d_ 98 +/- 13 nM), but severely attenuated. At a concentration of 3 uM, BVM only partly inhibited [^3^H]-BMS-977660 (BMS-‘176*). Total radiolabel binding to A364V was low; a reliable Kd could only be determined for BMS-955176.

**Table 4:** Affinities of BMS-955176 and BVM toward HIV-1 VLPs.

**VLP**	**BVM**				**BMS-955176**				**BVM/BMS -955176 K_d_ ratio**
	**K_d_ (nM)**	**SD**	**N**	**FC *vs*. WT**	**K_d_ (nM)**	**SD**	**N**	**FC *vs*. WT**	
WT	5.4	2.6	5	1.0	3.2	0.6	5	1.0	1.7
V362I	15	0.6	3	2.9	4.3	2.0	3	1.3	3.5
V370A	49.2	12.5	5	9.1	6.5	2.2	5	2.0	7.5
ΔV370	259	131	5	48	33.6	5.9	5	10	7.7
A364V	*				98	13	3	31	8.0
**VLP**	**BMS-1**			
	**K**_**d**_ **(nM)**	**SD**	**N**	**FC *vs*. WT**
WT	25.4	3.5	3	1.0
V362I	49	15.5	3	1.9
V370A	177	26.0	3	7.0
ΔV370	n.d.	n.d.	n.d.	n.d.
A364V	n.d.	n.d.	n.d.	n.d.

BVM, BMS-1 and BMS-955176 K_d_ values determined by competition displacement of [^3^H]-BMS-977660 (BMS-‘176*) as described in Materials and Methods.

* = Displacement by BVM of binding of [^3^H]-BMS-977660 to A364V too weak for reliable determination of Kd. Representative examples of competition displacement assay results are shown in S4 Fig. n.d. = not determined.

An adaptation of the binding assay was used to measure the kinetics of MI dissociation, as has been described for the determination of kinetics of dissociation of [3H] HIV integrase inhibitors from HIV-1 integrase.[65] Pre-formed MI/VLP complexes were treated with a large molar excess of a competitor MI, and the kinetics of dissociation followed over time. Representative examples of dissociation data are shown in S5 Fig, with data tabulated in Table 5. Dissociation half-lives for BVM and BMS-955176 were determined using their related C20:C29 double bond reduced tritiated derivatives, [3H] BMS-885221 ([3H BVM*) and [3H] BMS-977660 ([3H ‘176*), whose antiviral profiles are identical to BVM and BMS-955176, respectively. Dissociation half-lives for [3H]-BVM* and [3H]-176* were similar for WT VLP (41 vs. 51 minutes, Table 5). However, [3H]-BVM* dissociates rapidly from V370A and ΔV370 VLPs (≤ 3 minutes) and with an intermediate rate from V362I. By contrast, [3H]-176* displays similar dissociation kinetics towards all four VLPs. Dissociation of [3H]-176* from A364V was difficult to measure due to the low value of specific binding: T_1/2_ was rapid (< 2 minutes).

**Table 5:** Dissociation of BMS-176* and BVM* from HIV-1 VLPs.

	[^3^H]-BVM*			[^3^H]-BMS-176*			176*/’BVM* T_1/2_ ratio		Antiviral EC_50_, nM^a^		BVM/BMS-955176 MC EC_50_ ratio
VLP	T_1/2_ min	SD	N	T_1/2_ min	SD	N			BVM	BMS-955176	
WT	41	11	6	51	18	7	1.2		10.2	1.9	5.3
V362I	23	8	2	45	17	5	2.0		74	4.3	17
V370A	≤ 2.9	0.8	3	36	16	5	>12		553	2.7	205
ΔV370	≤ 2.4	0.8	2	46	13	5	>19		>10,000	13	>769

^a^ MC assay, from Table 1; ([^3^H BVM* and ^3^H-‘176* = C20 double bond reduced forms of BVM and BMS-955176; SD = standard deviation.

Rates of dissociation of BVM from V370A, V362I and ΔV370 were >12, 2.0 and >19-fold faster, respectively, compared to BMS-955176 (Table 5). This is similar in magnitude to the decreased affinities of BVM for these VLPs (9.1-, 3.5- and 48-fold, respectively, as compiled in Table 4). The antiviral potencies in cell culture toward the viruses with these polymorphs share the same trend as the affinity and off rate data: when compared with BVM, BMS-955176 binds with higher affinity and dissociates more slowly from the polymorphic VLPs, a result which is qualitatively correlated with its improved ability to inhibit replication of the cognate polymorphic viruses (Table 1). Interestingly, while BMS-955176 affinity (Table 4) and dissociation rates (Table 5) for WT, V362I and V370A are correlated (similar Kd values, similar dissociation half-lives), thus indicating that affinity is mainly driven by dissociation rates, affinity of BMS-955176 toward ΔV370 is reduced 10-fold as compared to WT (Table 4), though the dissociation rate is reduced by only 1.1-fold. This may indicate that reduced BMS-955176 affinity toward ΔV370 is due to a slower rate of association or possibly more complex multi-step binding kinetics, as has been observed for HIV-1 integrase strand transfer inhibitors.[66] This slower association rate implies a less pre-organized binding site, hindering the association of the ligand to its binding site, a point later addressed in the Discussion section.

### Modeling CA/SP1 cleavage rates as a function of Gag polymorph and MI

Biochemical studies of rate constants of polymorphic cleavages (Table 3), on one hand, and the binding affinities of MIs (Table 4), on the other hand, indicate that there is a qualitative relationship of each to the efficacy of a given MI to inhibit viral replication of a given polymorphic virus. From cellular assays, a plateau in inhibition (MPI values of <100%) suggests an escape mechanism that appears to contribute to reduced efficacy of a given MI towards different polymorphic viruses. Here, a model integrates both biochemical and cellular data to provide a more quantitative estimation of MI inhibition of CA/SP1 cleavage, and thus formation of mature viruses *in vivo*. The model (detail in Materials and Methods) has two terms which describe the observed rate of cleavage (k_clv,ob_) at CA/SP1 by HIV-1 protease in the presence of MIs.

$$k_{clv,ob}=\frac{k_{1}\;*\;K_{d}}{[MI]+K_{d}}+\frac{k_{2}\;*\;[MI]}{[MI]+K_{d}}$$

The first term describes the cleavage of the immature virus in the unbound state (B) (Fig 5). This term incorporates the innate cleavage rate constant k_1_ for different polymorphs and the concentration of the MI and its affinity (K_d_) for that polymorph. This is straightforward, and in accord with a simplified model (referred to here as model 1) in which only unbound state (B) is subject to protease cleavage. However, the observation of incomplete inhibition in antiviral assays (Table 2, MPI <100%) and the time-dependent loss in inhibition in *in vitro* cleavage assays (Fig 8) points to some degree of escape from inhibition as a function of both MI and polymorph, despite saturating concentrations of MI (Table 2, MPI <100%). To explicitly account for these observations, a second term was incorporated into the model for the rate of cleavage of immature virus in the MI-bound state (A), which is cleaved with rate constant *k*_2_, unique for each MI and polymorph. Essentially, the addition of this second term puts a cap on the maximal degree of inhibition of cleavage (referred to here as Model 2). Model 2a uses MPI values from the MC antiviral assay, with the concept that viral escape in the multiple cycle infection assay is a more realistic representation of clinical HIV replication (multiple cycle), as compared to the situation in the SC assay (Model 2b). An example of this approach is provided by BVM inhibition of the V370A virus where the antiviral MC MPI is 65.4%. This indicates that the fractional degree of cleavage arising from the bound form (A) is ((100–65.4)/100) = 0.345 of that arising from the unbound form (B). Thus the rate constant *k*_*2*_ = 0.345*k_1_.

**Fig 9:**
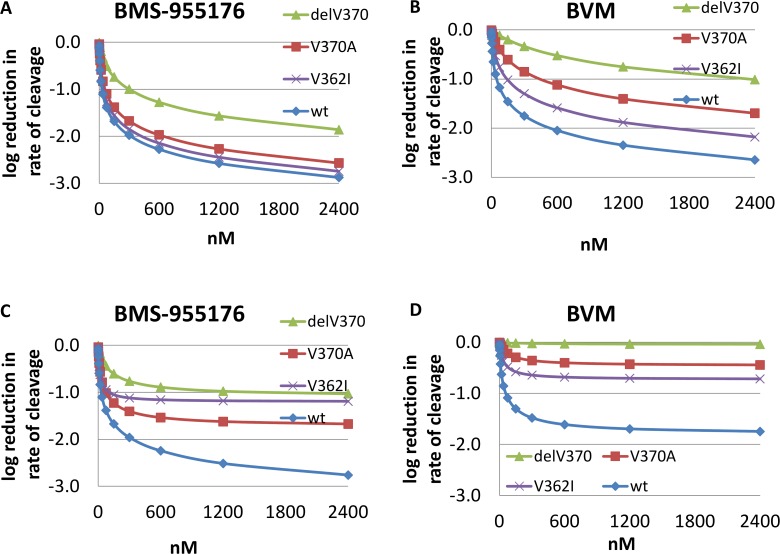
Modeling of rate of cleavage of WT, V362I, V370A and ΔV370 Gag VLPs in the presence of MIs. Simulation of log_10_ reduction in the observed rate of cleavage (k_*clv*,*ob*_) as a function of MI concentration. Upper panel: model 1, affinity and cleavage data, MI-unbound VLP (*k*_*1*_ term only); Lower panel: model 2a, affinity, MI-unbound VLP (*k1* term) plus (MI-bound VLP term *k2* (derived from incorporating antiviral MPI values from the multiple cycle assay). A) and C) BMS-955176, B and D) BVM.

Model 1 and 2a reductions in the rates of cleavage of WT, V370A, V362I and ΔV370 by BMS-955176 and BVM are depicted in Fig 9 as log_10_ reductions in cleavage rates vs. the uninhibited (no MI) control.

The upper panels (Fig 9A and 9B) were modeled with only the first term included (biochemical data only, model 1), while the lower panel was modeled with both terms included (biochemical and MC antiviral MPI data, model 2a). A key result for model 1 is that based solely on biochemical data, its estimation is in rough alignment with the antiviral results for these variants. A key result of model 2a (lower panel) is that there is a plateau in the degree of inhibition that depends on MI and polymorph, a direct result of the inclusion of the antiviral MPI data, bringing model 2a into closer alignment with the antiviral MPI data.

A quantitative comparison between BMS-955176 and BVM can be made from the modeling approaches at a selected MI concentration, for example at 300 nM MI (reductions spanning the entire range of concentrations are plotted in Fig 9, and tabulated in S1 and S2 Tables). At this concentration, BMS-955176 log_10_ reductions in WT virus (from the MC MPI data alone), log_10_ reductions in WT VLP cleavage rates (from model 1) and log_10_ reductions in WT VLP cleavage rates from model 2a are < -2.00 log_10_, -1.98 log_10_ and -1.96 log_10_ (Table 6), respectively. For BVM, these values are -1.80 log_10_, -1.75 log_10_ and -1.48 log_10_, respectively. Differences among the methods for the two MIs are somewhat larger for V362I (BMS-955176: -1.20/-1.85/-1.12 *vs*. BVM: -0.73/-1.30/-0.64), and V370A (BMS-955176: -1.73/-1.67/-1.41 *vs*. BVM: -0.46/-0.85/0.36, respectively). Log_10_ calculated reductions by BMS-955176 toward ΔV370 (BMS-955176: -1.09/-1.00/-0.76) are lower, as compared to wt and V370A, in accord with clinical results for subtype C viruses,[43] while values for BVM indicate essentially no antiviral activity (-0.04/-0.33/-0.02) toward the ΔV370 polymorph.

**Table 6:** Modeling effects of 300 nM MI on log_10_ reduction in rate of CA/SP1 cleavage/predicted reduction in viral RNA.

			virus or VLP CA/SP1 cleavage, (log_10_ reductions)		
Maturation Inhibitor	Model	WT	V370A	V362I	ΔV370
**BMS-955176**	multiple cycle antiviral assay, maximal inhibition	< -2.00	-1.73	-1.20	-1.09
**BMS-955176**	Model 1 (no escape mechanism)	-1.98	-1.67	-1.85	-1.00
**BMS-955176**	Model 2a (contains MC MPI values)	-1.96	-1.41	-1.12	-0.76
**BMS-955176**	single cycle antiviral assay, maximal inhibition	-1.15	-0.64	n.d.	-0.27
**BMS-955176**	Model 2b (contains SC MPI values)	-1.10	-0.61	n.d.	-0.23
**BMS-955176**	Clinical VLR	-1.75	-1.71		-1.35
					
**BVM**	multiple cycle antiviral assay, maximal inhibition	-1.80	-0.46	-0.73	-0.04
**BVM**	Model 1 (no escape mechanism)	-1.75	-0.85	-1.30	-0.33
**BVM**	Model 2a (contains MC MPI values)	-1.48	-0.36	-0.64	-0.02
**BVM**	single cycle antiviral assay, maximal inhibition	-0.71	-0.08	n.d.	0.06
**BVM**	Model 2b (contains SC MPI values)	-0.72	-0.07	n.d.	0.04
**BVM**	Clinical VLR	-1.26	-0.21		

Modeled reduction in CA/SP1 cleavage rates, and thus HIV-1 log_10_ RNA, at 300 nM MI; WT = wild type; in the models V362I and V370A serve as a comparators for reported substitutions which reduce BVM susceptibility toward polymorphic subtype B viruses, while ΔV370 serves as a comparator for non-subtype B viruses.[43] BMS-955176 clinical Ph2a data: 40 mg dose, maximal median HIV-1 log_10_ RNA reduction as reported for subtype B WT and polymorphs [42, 67]) and subtype C viruses.[43] BVM clinical Ph2a data at 250–400 mg doses: mean HIV-1 log_10_ RNA reduction as reported for patients achieving C_trough_ of > 20μg/mL BVM[18, 68, 69]. VLR = viral load reduction. Models as described in Fig 5 and materials and methods.

Model 1 wt reductions for BVM (-1.75), are similar to that from the antiviral MPI data (-1.80). Model 1 V362I, V370A and ΔV370 predictions are somewhat larger vs. antiviral data. By comparison, the inclusion of MC antiviral MPI data (model 2a) results in lower predicted log_10_ reductions for wt and polymorphs, in line with the antiviral data for BVM.[18, 68]

**Fig 10:**
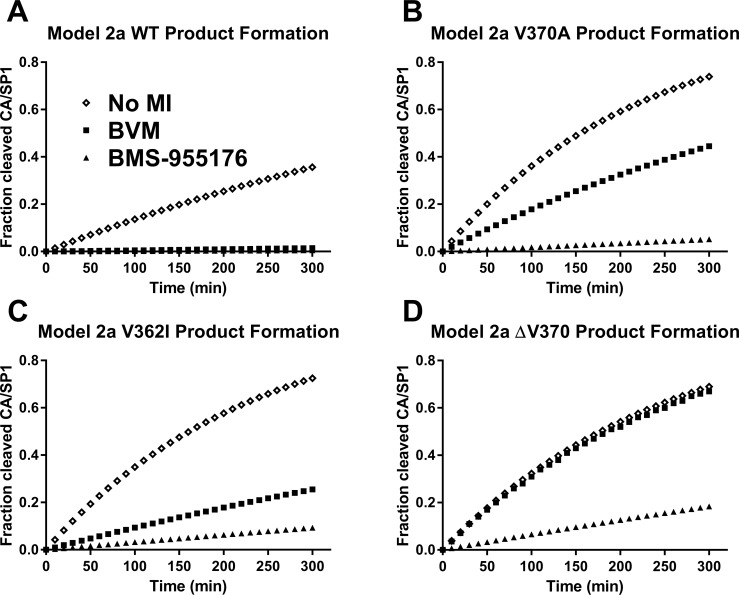
Modeling of the rate of CA/SP1 cleavage of HIV-1 WT, V370A, V362I and ΔV370 VLP in the presence of 300 nM MI. Modeled fractional rate of production of SP1 peptide from Gag VLP cleavage using model 2a at 300 nM MI, as noted in text; no MI: diamonds; BVM: squares; BMS-955176: triangles; y-axis: fraction of CA/SP1 cleavage is a surrogate for production of mature virus, as indicated in Fig 5; A) WT; B) V370A; C)V362I; D) ΔV370

Table 6 also contains calculated log_10_ reductions for wt, V362I, V370A and ΔV370, using a modification of model 2 in which MPI values are taken from the SC assay (model 2b, no term for *k*_*2*_). In these cases, model 2b gives similar results to those taken directly from log_10_ viral reductions calculated directly from the SC antiviral MPI values (as to be expected given the weight of the SC MPI-derived term in the equation which dominates the response over that of the biochemical-only model 1), and under-predicts the clinical responses. Model 2a time courses for the appearance of cleavage product SP1 peptide by HIV-1 protease for WT, V362I, V370A and ΔV370 VLP, and inhibition profiles by 300 nM BVM or BMS-955176, are shown in Fig 10.

**Fig 11:**
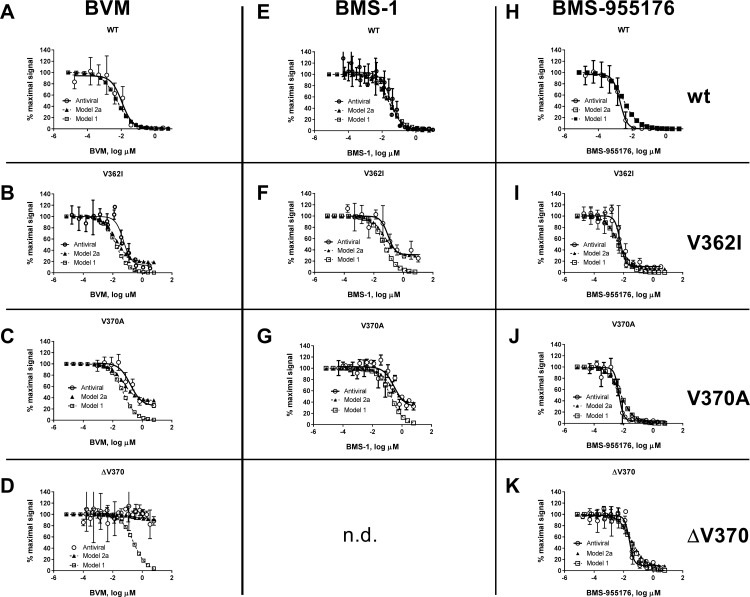
Calculated vs. observed antiviral responses of wt and polymorphic viruses to BVM. **BMS-1 and BMS-955176.** Antiviral dose response curve for inhibition of HIV-1 wt (A, E, H) and Gag polymorphic variants V362I (B, F, I) and V370A (C, G, J) by MIs BVM (column 1), BMS-1 (column 2) and BMS-955176 (column 3); antiviral data (open circles, solid line) are the mean of at least 3 experiments normalized to no response = 100% activity. Modeled data is normalized to calculated rates of CA/SP1 cleavage (*k*_clv,obs_) for model 1 (open squares, dotted line) and model 2a (closed triangles, dotted line).

As further detailed in S1 Table (model 1, no MPI data included), 300 nM BMS-955176 reduces the rate of cleavage of WT, V362I, V370A and ΔV370 by 95, 71, 47 and 10-fold, respectively. BVM is effective at reducing the rate of WT cleavage (57-fold), less effective toward V362I (20-fold), much less effective toward V370A (7.1-fold), and ineffective toward ΔV370 (2.2-fold). Model 2a (S2 Table) indicates that 300 nM BMS-955176 reduces the rate of cleavage of WT, V362I, V370A and ΔV370 by 91-, 13-, 25- and 6-fold, respectively. By comparison, model 2a indicates that while BVM is effective at reducing the rate of WT cleavage (30-fold), it is far less effective toward V362I (4.4-fold) and ineffective toward V370A (2.3-fold) and ΔV370 (1.1-fold). Another way to visualize the results is to compare antiviral dose-response curves to those generated from the models across all concentrations. Plateaus in antiviral inhibition are apparent, particularly for BVM and BMS-1 toward polymorphic variants, as noted in Table 2. This is shown in Fig 11, which displays the antiviral dose-responses (MC assay) for combinations of BVM, BMS-1 and BMS-955176 toward wt, V362I, V370A and ΔV370 viruses, compared to the calculated values from models 1 and 2a (exception: the combination of BMS-1 with ΔV370 was not performed). The results illustrate that the antiviral data is in better alignment with model 2a compared to model 1. The data also highlight that binding per se is insufficient to result in a complete antiviral effect (MPI values of 100%) for several combinations of MIs and polymorphic variants, for example for BVM and BMS-1 toward V362I, V370A and ΔV370, despite binding, (Fig 11 and Table 4).

### Comparison of modeling results to viral load reduction responses in clinical studies of BMS-955176 and BVM

Clinical viral load reduction (VLR) data from BMS-955176[41–43, 70] and BVM clinical trials[18, 28, 68] are shown in Table 6. Clinical VLR reductions were compared to reductions in rates of CA/SP1 cleavage using the different models at a concentration of 300 nM (fold reduction values relative to no MI added to each particular virus). This concentration of MI was chosen for the comparison for two reasons. First, BVM trough concentrations of >20 μg/mL were associated with the best clinical responses[18, 28, 68] and based on a BVM antiviral serum shift of 130-fold[35, 36, 40] the implied free concentration of 20 μg/ml BVM is 263 nM. Similarly, the clinical response of BMS-955176 in a 10 day Ph2a study reached a plateau at C_24_ exposures between 713 and 1289 nM[67] (mean = 1521 nM), implying a mean free concentration (based on a reported free fraction of 0.14)[40] of 213 nM. Thus, modeling was compared at 300 nM for both MIs.

The maximal median decline for subjects having a WT genotype at 40 mg QD dosing by BMS-955176 in a Ph2a POC 10 day monotherapy study was (-1.75) log_10_ (Table 6).[41] This value is slightly less than both the model 1 and 2a values (~-2 log_10_), and less than the value directly calculated from the MPI in the MC assay (< -2.00 log_10_). With respect to subjects harboring Gag polymorphisms (Gag amino acids 362, 364, 370, 371) at a dose of 40 mg BMS-955176, a comparison can be made to V370A, with V370A acting as a kind of surrogate for such polymorphisms (there is currently no available data breaking out patient responses to individual polymorphic viruses). Model 1 values for polymorphs V370A and V362I (-1.67 and -1.85 log_10_, respectively), or the values directly calculated from the MPI in the MC assay (-1.73 and -1.20 log_10_), are similar to the clinical response of BMS-955176 reported for polymorphs (-1.71 log_10_), while the projected values from model 2a for V370A and V362I, incorporating the MC MPI data (-1.41 and -1.12 log_10_, respectively), are somewhat lower than reported for subjects with these polymorphic genotypes. The model 2b V370A value (-0.61 log_10_), using single cycle MPI data, greatly underestimates the clinical result for subjects harboring polymorphic viruses, thus suggesting that SC MPI values are likely too stringent, leading to an under-estimation of clinical responses (Table 6).

**Fig 12:**
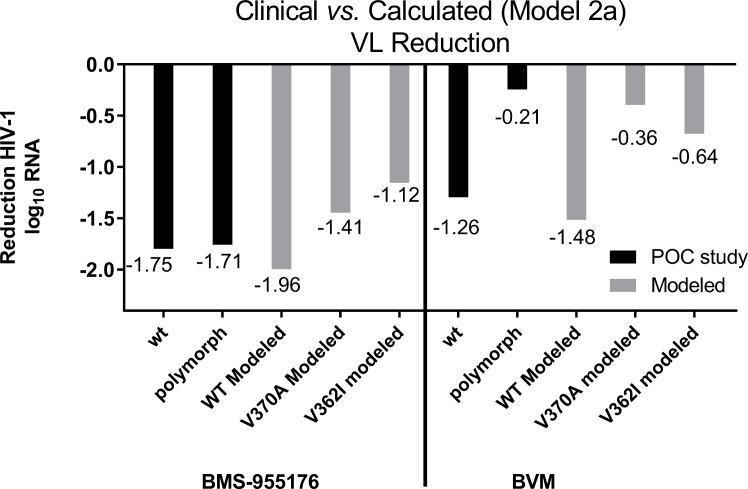
Calculated (Model 2a) *vs*. observed responses for BMS-955176 and BVM vs. clinical results. Model 2a reduction in rate of CA/SP1 cleavage of WT, V362I and V370A (surrogates for polymorphic viruses, as described in the text) at 300 nM MI, *vs*. observed reduction in HIV-1 log_10_ c/mL RNA in proof of confidence (POC) studies; WT = wild type; BMS-955176 polymorphic clinical subtype B = changes at positions Gag 362, 369, 370 or 371; BVM polymorphic clinical = changes at positions Gag 369, 370 or 371; BMS-955176 clinical POC study, 40 mg maximal median HIV-1 log_10_ RNA reduction; BVM clinical POC study, 250–400 mg.

For BVM, the mean decline for those subjects achieving trough concentrations of >20 μg/mL[28, 29] with a WT genotype at 250–400 mg QD in a Ph2 14 day monotherapy POC study was -1.26 log_10_.[18] This value is lower than both the WT model 1 calculated decline (-1.75 log_10_) or the value directly calculated from the MPI in the MC assay (-1.80 log_10_). The model 2a value (-1.48 log_10_) is closer to the clinical data. A mean -0.21 log_10_ decline was noted in subjects harboring Gag polymorphisms (Gag amino acids 369, 370, 371) at doses of 250–400 mg BVM,[68] which may be compared to the V370A and V362I polymorphic viruses used in this study. The model 1 BVM declines (-0.85 and -1.30 log_10_) over-predict the clinical response, while the projected declines calculated from the MPI values (solely from the MC assay) for these two variants (-0.46 and -0.73 log_10_) or model 2a (-0.36 and -0.64 log_10_), respectively, are in closer alignment for these types of polymorphic patient viruses (-0.21 log_10_). The calculated reductions in CA/SP1 cleavage rates for WT and polymorphic viruses V362I and V370A at 300 nM MI (Table 6) are compared in Fig 12. Overall, of the models, model 2a, incorporating MC assay MPI values, provides a better correspondence to both antiviral dose response curves (Fig 11) and clinical viral load reductions (Fig 12).

## Discussion

An early MI failed in the clinic due to inability to inhibit ~50% of viruses containing polymorphic variation in Gag near the site of MI action. The 2^nd^ generation MI, BMS-955176, is active toward these viruses. In this study we sought to understand the mechanistic origin for the improved antiviral activity of BMS-955176, and to model this behavior as a function of Gag polymorph cleavage rates, MI affinity and MI concentration, with consideration as to how this information relates structurally to MI binding. Such an approach may have utility in interpreting pre-clinical antiviral results and clinical data on MI action, and may also be helpful in the discovery of MIs with further improvements to potency and spectrum.

The higher affinity of BMS-955176 toward Gag polymorphs appears to be a predominant driver for better antiviral activity toward Gag polymorphs (both lower EC_50_ values as well as higher MPI values). Similarly, higher BMS-955176 affinity is apparently an important driver for the superior performance in *in vitro* cleavage assays. BMS-955176 inhibition is maintained against WT at all time points (4 hours), while BVM inhibition is lost over time. Consistent with the overall relationship, in a case where BMS-955176 has a phenotype of partial time-dependent inhibition (*in vitro* cleavage for ΔV370, Fig 8B), this was correlated to an elevated FC in antiviral assays (Table 1, 6.8-fold) and a reduced MPI value in the MC assay as compared to WT (93 *vs*. 100%). One of the features of the inter-relationships of the data is that K_d_ values are linearly correlated with both MPI values and FC antiviral EC_50_ values (R^2^ 0.96), with greater affinity providing higher MPI values and lower FC.

Antiviral and biochemical data were integrated into a model for calculating the reduction in the rate of cleavage of CA/SP1 by a given MI/polymorphic combination. Modeled reductions in rates of CA/SP1 cleavage by BMS-955176 and BVM were compared to antiviral data in cell culture and viral load reductions observed clinically with these MIs using several models, the most relevant model being one which incorporates both biochemical MI affinities for its Gag target, innate cleavage rates for the viruses and values for MPI from multiple cycle anti-viral data (Model 2a). At a dose of 40 mg QD BMS-955176 in a 10 day monotherapy POC study, the maximal median viral load declines for subjects having WT or polymorphic genotypes were -1.75 log_10_ and -1.71 log_10_, respectively, in alignment to values calculated from model 2a (wt: -1.96 log_10_, V370A: -1.41 log_10_, V362I: -1.12 log_10_). Similarly, at doses of 250–400 mg QD BVM in a Ph2a 14 day monotherapy study of subtype B patients, the mean viral load declines for subjects having a WT or polymorphic genotype were -1.26 log_10_ and -0.21 log_10_, respectively, in the range of values calculated from model 2a (wt: -1.48 log_10_, V370A: -0.36 log_10_).

These studies determined that *in vitro* inhibition of HIV-1 replication by early generation MIs BVM and BMS-1 does not always reach 100%. It should also be noted that for one polymorphic variant (ΔV370) BMS-955176 also does not always reach 100% inhibition as well; albeit to a significantly reduced degree. This observation with respect to early generation MIs was observed across polymorphs, and was correlated with a reduction in antiviral potency (increased fold change EC_50_ values) by a particular MI toward the particular virus containing that Gag polymorph. For example, in a multiple cycle assay, BVM maximally inhibits the replication of HIV-1 Gag V370A by 65.4%, and, in a single cycle assay, by 19%, exhibiting a 54-fold change in its multiple cycle EC_50_. These observations suggest that, depending on polymorph and MI, this phenomenon is analogous to one of partial antagonism. In seeking the mechanistic origins of this behavior we initially considered a simplified model for MI inhibition of CA/SP1 cleavage of viral particles in which cleavage only takes place in that fraction of particles not bound to the MI. Thus, CA/SP1 cleavage should continue apace on the MI-unbound particles at a rate determined by the steady state fraction of unbound MI. Because of this, model 1 places no upper limit on the degree of maximal inhibition: at saturating MI concentrations the fractional amount of unbound Gag will approach zero, and thus complete inhibition is to be expected. However, the antiviral phenotype of incomplete inhibition in cell culture at saturating BVM concentrations argues against this simple model, thus suggesting the need for a modification to the model to explicitly include a term which ultimately places an upper value on the degree of maximal inhibition. For this purpose we made use of the MC MPI values, which we interpret as a direct functional readout of viral escape from MI action in cell culture.

Parameterizing the biochemical-only model (model 1) required determination of the appropriate biochemical values for the innate rates of HIV cleavage and the affinities of MIs toward assembled Gag virus-like particles. These measurements were made by developing two assays. In the first, we made use of an LC/MS-based assay that directly measures CA/SP1 cleavage *vs*. time, thus providing rate constants for this process as a function of polymorph. These results showed that Gag polymorphic variants that are less susceptible to inhibition of replication by early generation MIs BVM and BMS-1 (Table 1) are cleaved 2.7–9.7-fold more rapidly than the WT (Table 3) and they correspondingly exhibit the most pronounced incomplete inhibition profiles (MPIs <100%) in antiviral assays (Table 2 and Fig 11). In a qualitative sense, poorer antiviral coverage of these polymorphs appears to be in part a consequence of poorer MI affinity for Gag, but also is a reflection of a lack of ability of BVM and BMS-1 to fully inhibit when bound, i.e., consistent with the proposed pathway in which cleavage occurs despite MI binding (*k*2-mediated, Fig 5). This results in what is in essence partial antiviral antagonism, as a function of MI and polymorph, which cannot be overcome by merely increasing MI concentration. V362I is more sensitive to BVM inhibition *vs*. V370A. Though superficially posing a challenge to a model in which efficacy of inhibition of CA/SP1 cleavage is entirely a function of cleavage rate, this is not the case for model 2a, where terms 1 and 2 of equation (see model for inhibition) also incorporate the K_d_ value for the binding of the MI. In this case BVM affinity for V370A is 9.1-fold poorer than wt, while BVM affinity for V362I is 2.9-fold reduced. This 3-fold higher affinity for V362I contributes, in part, to allowing BVM to maintain, albeit incompletely and right shifted, activity toward V362I, while losing activity toward V370A.

Mechanistically, what structural model might explain the result of escape from inhibition, despite binding? The following proposed model is based on a number of reported observations. First, NMR studies indicated that the superstructure around CA-SP1 in the region of MI binding (SP1) is in dynamic equilibrium between a random coil and an alpha helix.[71] In support of this dynamic equilibrium model, small changes to buffer and detergent alter the helicity of the SP1 region [72] while point mutations predicted to reduce helicity destroy particle production. [73–75] Earlier cryo-electron tomography work on immature particles found that the extension of SP1 from the C-terminal region of CA could be fitted as a six-helix bundle, leading to a proposal that cleavage at CA-SP1 acts as a molecular switch, facilitating the final conformational changes required for capsid rearrangement and core condensation. [76, 77]

A deeper structural understanding is now at hand with the report of a cryo electron tomography structure of the immature assembled Gag lattice at 3.9 angstrom resolution and a crystal structure reported at at 3.2 angstrom resolution [61, 62] The structures indicate that the CA-SP1 cleavage site is hidden within this 6-helix bundle, and protected from cleavage due to inaccessibility, a structural explanation for why cleavage at this site is the slowest of the Gag cleavages.[15] MI binding is suggested to rigidify the structure and likely shifts the equilibrium of the superstructure in favor of the 6-helix structure, thus reducing the propensity for unraveling and presentation of the cleavage site. This is in accord with a report that BVM binding increases the stiffness of immature virions.[78] The formation of a more ordered helical state as a consequence of MI binding in this region, shown by cross linking studies of BVM analogs at sequences overlapping or proximal to the CA-SP1 cleavage site, is also consistent with previous biochemical data on the effect of bevirimat on Gag processing, and with genetic data from resistance mutations.[60]

The results reported in this study are in alignment with these structural results and proposal for the role of polymorphic or MI resistance changes which increase cleavage site presentation. As compared to wt, the more rapid innate rates of CA/SP1 cleavage of certain polymorphs are therefore explainable as a reflection of a decrease in the stability or equilibrium concentration of the bundle, i.e., the inherently greater degree of disorder in the cleavage region allows for the presentation of the protease recognition site in its extended conformation a greater proportion of the time. The modeled biochemical and viral data, which showed improved inhibition of *in vitro* cleavage and higher maximal antiviral inhibition by BMS-955176 are consistent with a global explanation for the broader antiviral coverage of BMS-955176 *vs*. BVM: the increased affinity of BMS-955176 for its binding site increases the concentration and perhaps structural integrity of the quarternary structure of the assembled 6-helix bundle This thereby decreases dynamic fraying of the structure which would otherwise lead to protease cleavage.

With respect to the observed phenotype of partial antagonism by certain MI/polymorph combinations, the data suggests that binding in and of itself is not always sufficient to induce changes in the local geometry needed to completely prevent protease recognition of CA/SP1 and thereby completely block cleavage. This may be the case for V362I vs. V370A. While these two polymorphs are cleaved with similar rates (Table 3), they exhibit differing MPI values depending on MI. The wt MI-bound Gag structure is likely innately more ordered to begin with, while polymorphic variants, with greater innate flexibility and reduced local order, retain some bias in this direction, despite MI binding (Fig 5, pathway 2), rendering them partly susceptible to cleavage even in the MI-bound state. This suggests that depending on the particular effects induced on the local conformation by a given polymorphic change and the particular binding poise of an MI, the consequences of that binding may be only partially transmitted to the key conformation changes that are meaningful for antiviral activity, that of maintaining reduced access of the CA/SP1 cleavage site to protease. Thus, biochemically one observes a time dependence to the *in vitro* cleavage inhibition, while in antiviral assays, less than maximal antiviral inhibition. This is an escape mechanism.

At the structural level, the ability of the 2^nd^ generation MI BMS-955176 to induce greater protection from cleavage of polymorphs is possibly due to additional binding contacts within the Gag structure, reflected in its higher binding affinity, but a detailed explanation must await MI bound structures. Such binding presumably contributes to a greater stabilization of that local conformation (presumably increased helicity of SP1) which renders the system less sensitive to protease recognition/cleavage. Further, while the generality of the conclusion that faster innate rates of polymorphic cleavage are a reflection of greater flexibility and accessibility of the CA/SP1 site to protease recognition and cleavage seems therefore to be sound, further studies are needed to understand the structural details of MI binding, in particular to shed light on those cases where saturable binding is still not maximally productive (partial antagonism).

Given the similar dissociative off rates of BMS-955176 toward wt and ΔV370 VLPs, but the higher affinity toward wt, the calculated rate of association toward the ΔV370 variant is implied to be ~9-fold slower than wt (from consideration of a simple 1 step binding model (kon = koff x Kd). This slower on rate may reflect a more unstructured MI-unbound structure in the vicinity of the ΔV370 MI binding site (as compared to, for example, V370A, with a calculated relative on rate similar to wt). From the published structure, position 370 is at the end of the 6-helix bundle, so potentially deletions in this region introduce unzipping and greater local disorder, with such a disordered state obscuring the trajectory of MI binding, and thereby inducing an entropic penalty to binding.

While further work is clearly needed to more fully understand the relationship of modeled to antiviral and clinical results, the approach described herein to understand MI activity and mechanism should prove useful to potentially facilitate further improvements to MI potency and coverage.

## Supporting Information

S1 Table Model 1 Fold reduction in rate of cleavage (k_*clv*,*ob*_) as a function of MI concentration. Model 1 results: data modeled with biochemical data only (cleavage rate constants and MI affinities).
(TIF)

S2 Table Model 2a Fold reduction in rate of cleavage (k_*clv*,*ob*_) as a function of MI concentration. Model 2a results: data modeled with biochemical data (cleavage rate constants and MI affinities) and also included a term for rate constant, k_2_, derived from MC antiviral MPI data (Table 2 and Materials and Methods).
(TIF)

S1 Fig Recovery of MI in 10% FBS vs. PBS as a function of time and concentration. Experiments performed with 3 and 6 μM MI dissolved in 10% FBS media vs. PBS buffer for up to 4 days. Samples were centrifuged at 14 x g for 10 minutes and supernatant subsequently analyzed by LC/MS for concentration of soluble MI.
(TIF)

S2 Fig Rate constants for the cleavage at CA/SP1. Rate constants were further quantified by measuring rates for selected VLPs (WT, ΔV370 and A34V) as a function of HIV-1 protease concentration (Supplementary Fig 2A). The time course for cleavage is nearly linear for WT Gag out to 240 minutes. A degree of non-linearity is observed for the ΔV370 variant at higher protease concentrations and longer cleavage times. This is more pronounced for A364V, where nonlinearity is observed within 30 minutes, likely due to substrate depletion. Supplementary Fig 2B compares the relative production of SP1 for the 3 Gag variants at a single protease concentration (270 nM), while Supplementary Fig 2C shows the linearity of the first order rate constants, providing 2^nd^ order rate constants for the production of SP1 for these three variants (Supplementary Fig 2D). As shown in Supplementary Fig 2D the 2^nd^ order rate constant for the production of SP1 from ΔV370 is 2.2-fold faster than WT, and that of A364V is 9.1-fold faster than WT. The result of this more complete kinetic profile is in alignment with that of the first order rate constants measured using the single 270 nM HIV-1 protease concentration (Table 3, text).
(TIF)

S3 Fig SP1 product appearance curves for cleavage of HIV-1 VLPs by HIV-1 protease. Progress curves for the generation of AM peptide (SP1) as a function of time at 270 nM HIV-1 protease; data in Table 3.
(TIF)

S4 Fig Competition Displacement of Binding of [^3^H]MI. One experiment showing representative examples of displacement of binding of [3H] BMS-977660 ([3H ‘176*) to WT, V370A, V362I and A364V VLPs by BVM, BMS-1 and BMS-955176. Error bars (N = 3) shown.
(TIF)

S5 Fig Representative dissociation curves of MIs from HIV-1 VLPs. Representative dissociation kinetics of MIs towards WT, V370A and ΔV370 VLPs, dissociation half-lives for BVM and BMS-955176 were determined using their related C20:C29 double bond reduced tritium derivatives, [3H] BMS-885221 ([3H BVM*) and [3H] BMS-977660 ([3H ‘176*), whose antiviral profiles are identical to BVM and BMS-955176, respectively.
(TIF)
