# Supplementary figures and images for: Mechanistic Studies and Modeling Reveal the Origin of Differential Inhibition of Gag Polymorphic Viruses by HIV-1 Maturation Inhibitors
Source: PLoS Pathog. 2016 Nov 28;12(11):e1005990. doi: 10.1371/journal.ppat.1005990 (PMC5125710; doi:10.1371/journal.ppat.1005990)

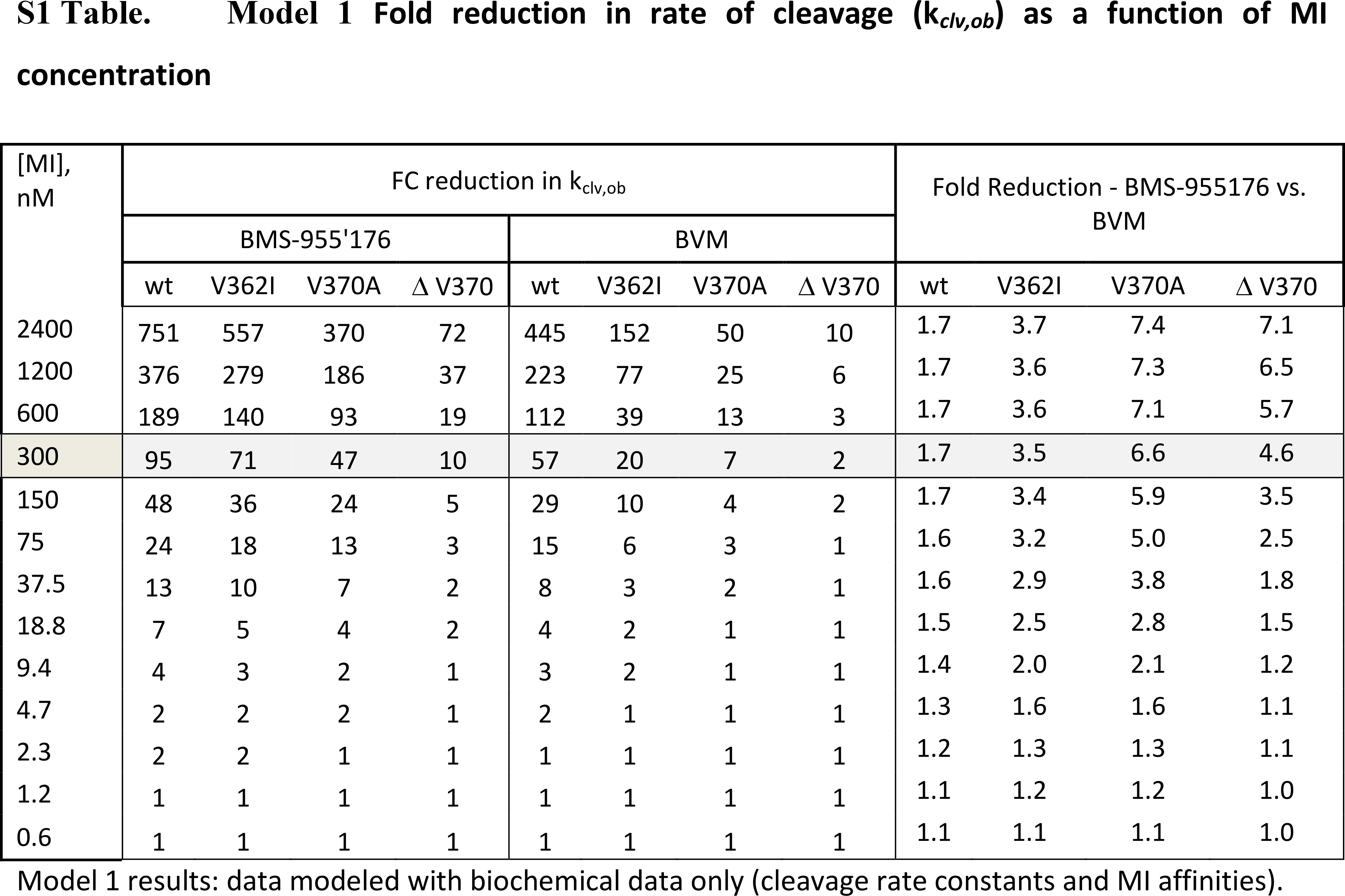

Supplement: S1 Table — Model 1 results: data modeled with biochemical data only (cleavage rate constants and MI affinities). (TIF) [file ppat.1005990.s001.tif]

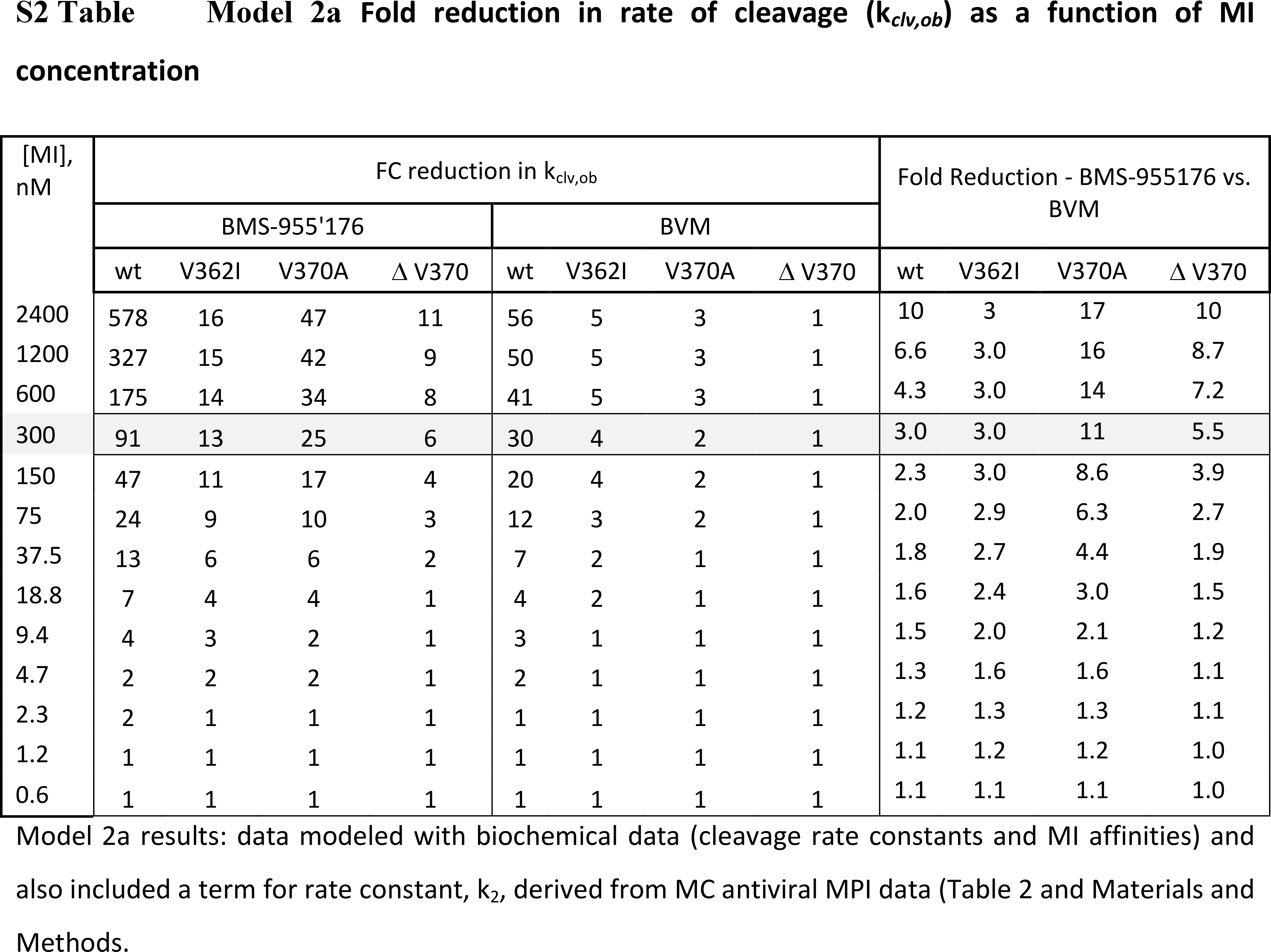

Supplement: S2 Table — Model 2a results: data modeled with biochemical data (cleavage rate constants and MI affinities) and also included a term for rate constant, k2, derived from MC antiviral MPI data (Table 2 and Materials and Methods). (TIF) [file ppat.1005990.s002.tif]

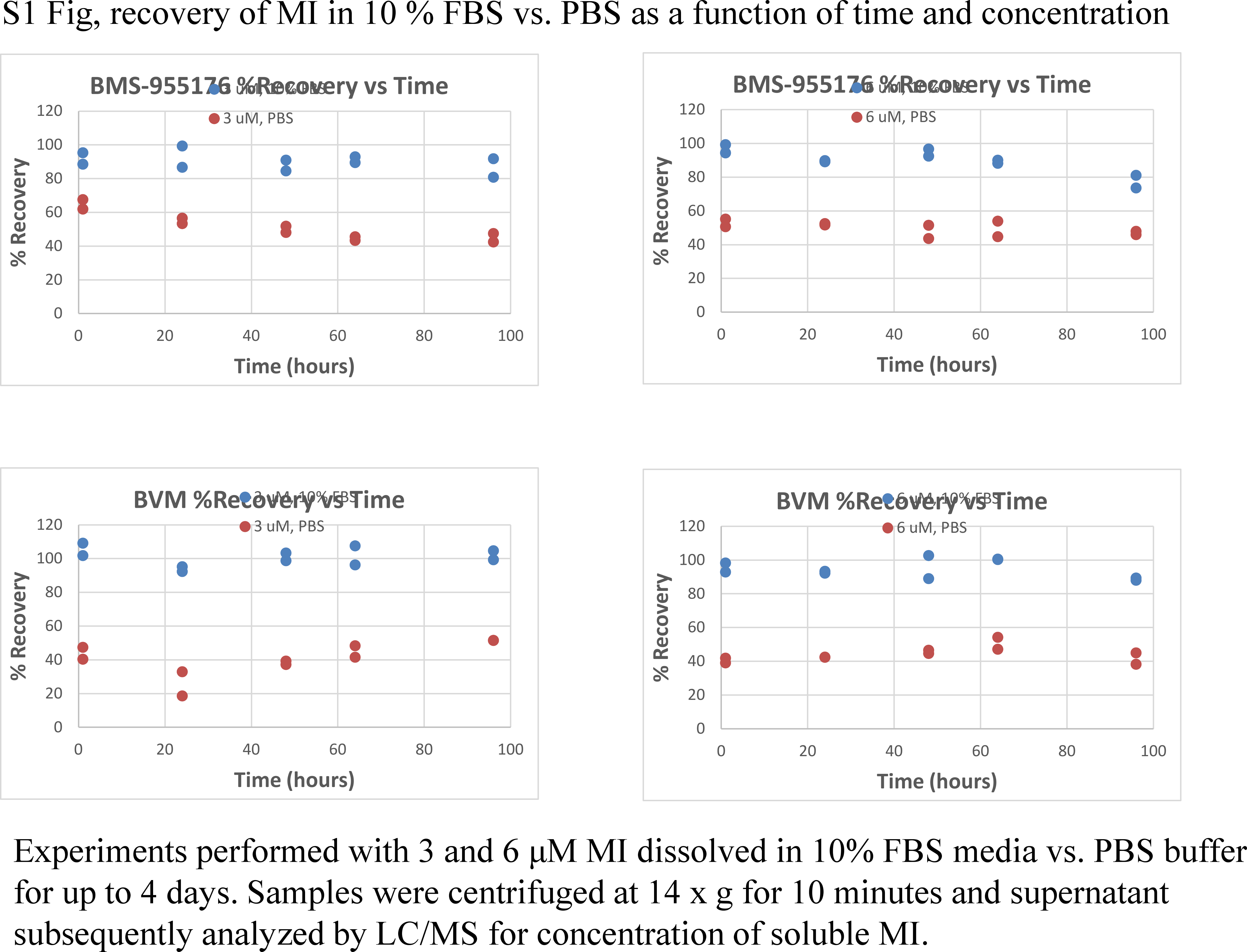

Supplement: S1 Fig — Experiments performed with 3 and 6 μM MI dissolved in 10% FBS media vs. PBS buffer for up to 4 days. Samples were centrifuged at 14 x g for 10 minutes and supernatant subsequently analyzed by LC/MS for concentration of soluble MI. (TIF) [file ppat.1005990.s003.tif]

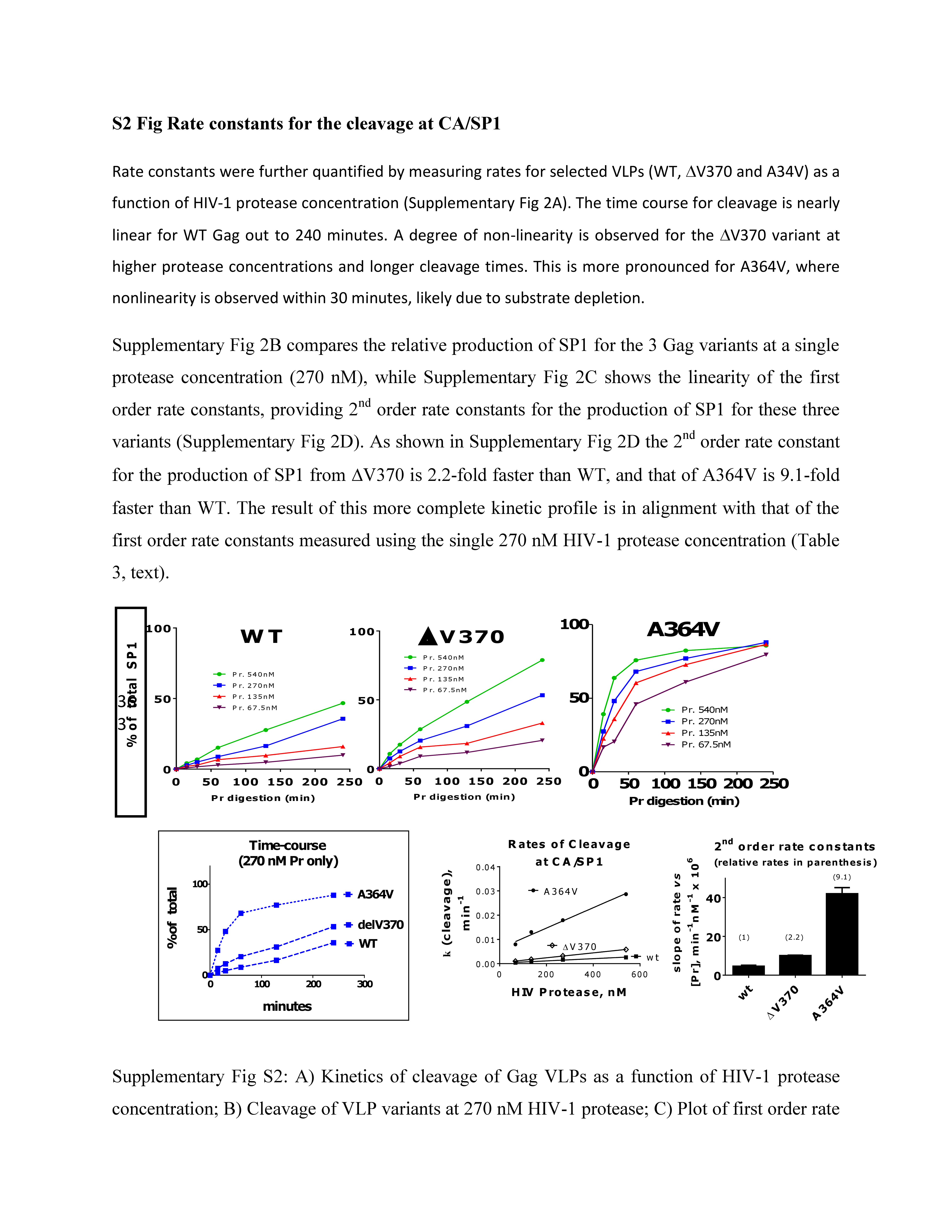

Supplement: S2 Fig — Rate constants were further quantified by measuring rates for selected VLPs (WT, ΔV370 and A34V) as a function of HIV-1 protease concentration (Supplementary Fig 2A). The time course for cleavage is nearly linear for WT Gag out to 240 minutes. A degree of non-linearity is observed for the ΔV370 variant at higher protease concentrations and longer cleavage times. This is more pronounced for A364V, where nonlinearity is observed within 30 minutes, likely due to substrate depletion. Supplementary Fig 2B compares the relative production of SP1 for the 3 Gag variants at a single protease concentration (270 nM), while Supplementary Fig 2C shows the linearity of the first order rate constants, providing 2nd order rate constants for the production of SP1 for these three variants (Supplementary Fig 2D). As shown in Supplementary Fig 2D the 2nd order rate constant for the production of SP1 from ΔV370 is 2.2-fold faster than WT, and that of A364V is 9.1-fold faster than WT. The result of this more complete kinetic profile is in alignment with that of the first order rate constants measured using the single 270 nM HIV-1 protease concentration (Table 3, text). (TIF) [file ppat.1005990.s004.tif]

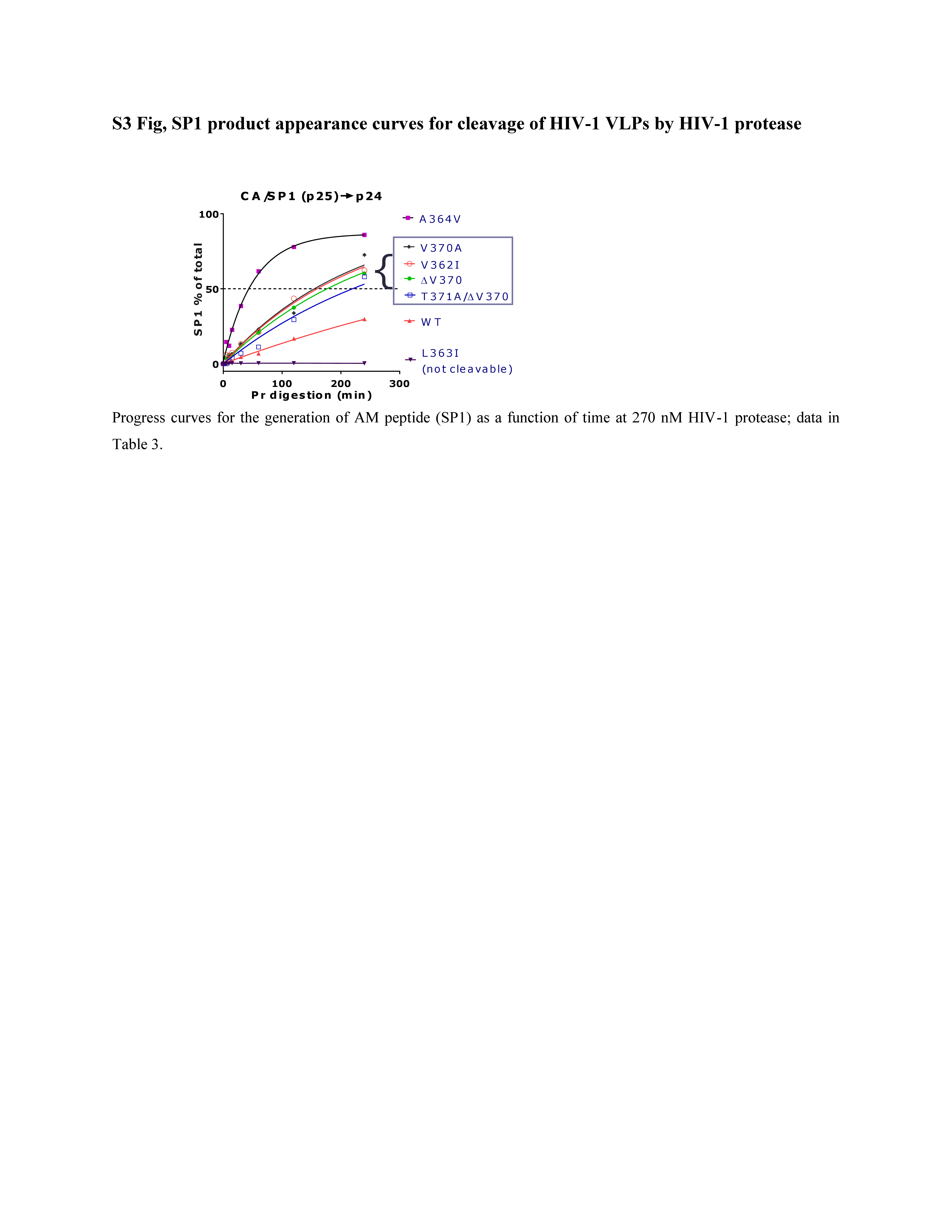

Supplement: S3 Fig — Progress curves for the generation of AM peptide (SP1) as a function of time at 270 nM HIV-1 protease; data in Table 3. (TIF) [file ppat.1005990.s005.tif]

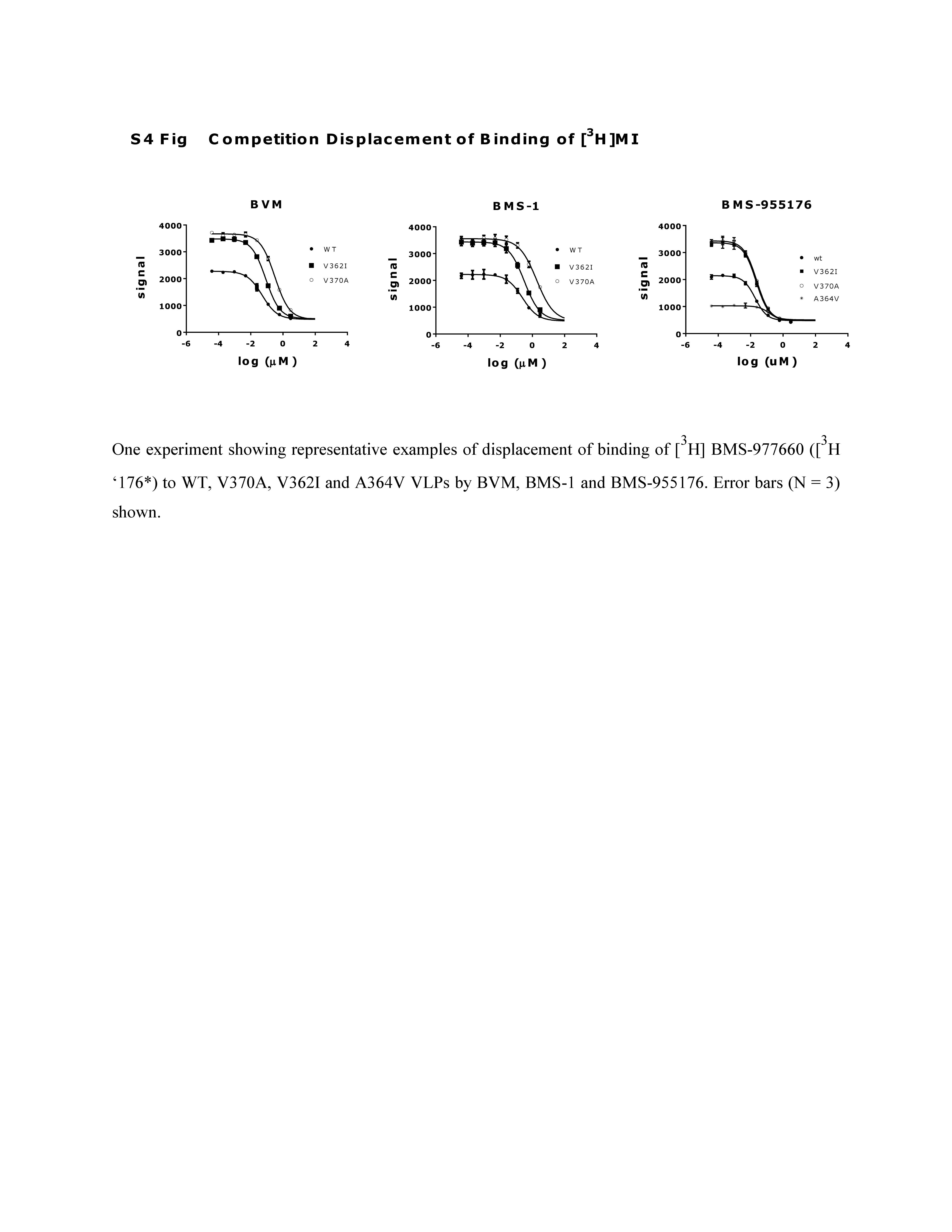

Supplement: S4 Fig — One experiment showing representative examples of displacement of binding of [3H] BMS-977660 ([3H ‘176*) to WT, V370A, V362I and A364V VLPs by BVM, BMS-1 and BMS-955176. Error bars (N = 3) shown. (TIF) [file ppat.1005990.s006.tif]

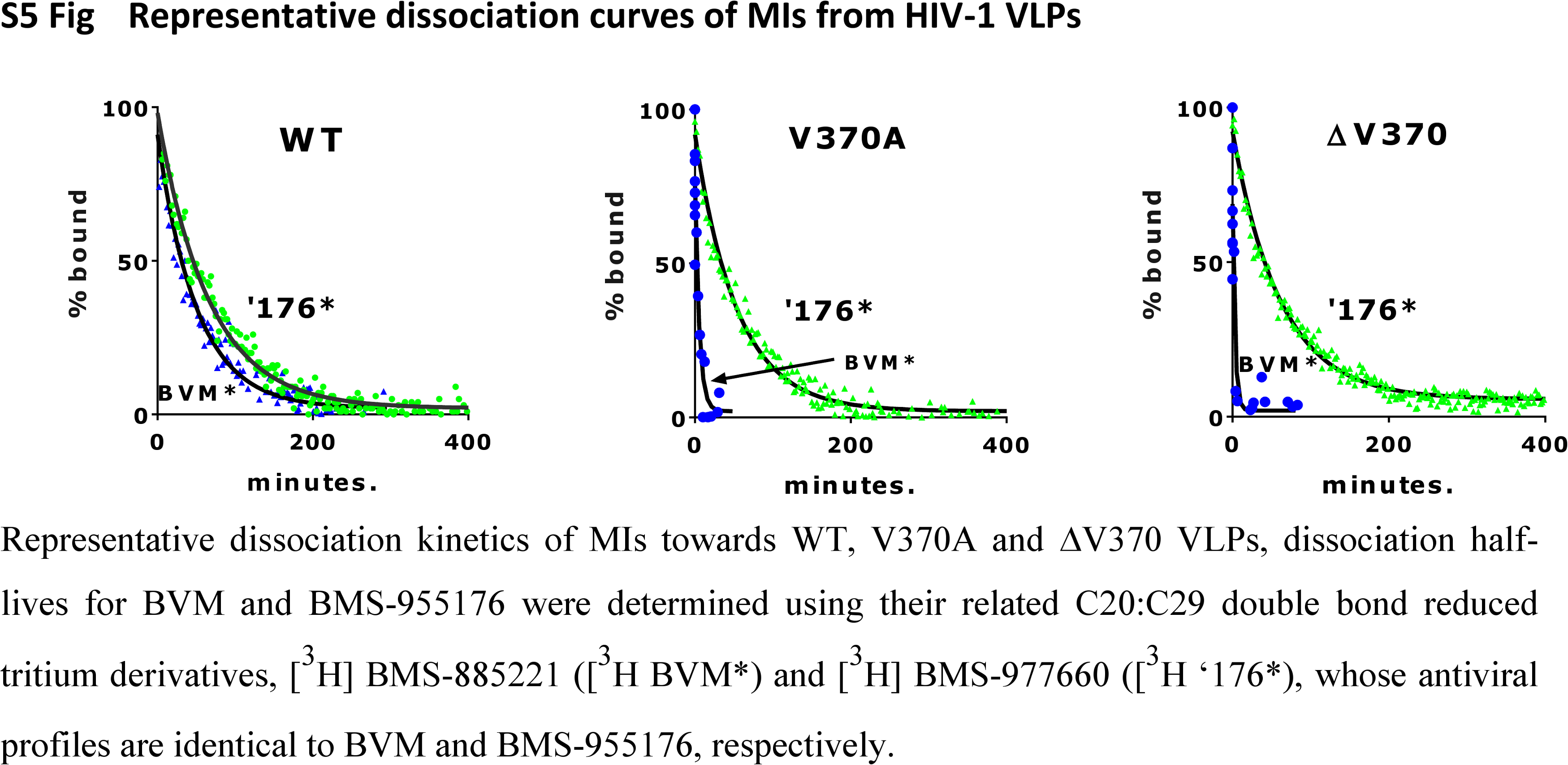

Supplement: S5 Fig — Representative dissociation kinetics of MIs towards WT, V370A and ΔV370 VLPs, dissociation half-lives for BVM and BMS-955176 were determined using their related C20:C29 double bond reduced tritium derivatives, [3H] BMS-885221 ([3H BVM*) and [3H] BMS-977660 ([3H ‘176*), whose antiviral profiles are identical to BVM and BMS-955176, respectively. (TIF) [file ppat.1005990.s007.tif]
